# Physician-directed genetic screening to evaluate personal risk for medically actionable disorders: a large multi-center cohort study

**DOI:** 10.1186/s12916-021-01999-2

**Published:** 2021-08-18

**Authors:** Eden V. Haverfield, Edward D. Esplin, Sienna J. Aguilar, Kathryn E. Hatchell, Kelly E. Ormond, Andrea Hanson-Kahn, Paldeep S. Atwal, Sarah Macklin-Mantia, Stephanie Hines, Caron W.-M. Sak, Steven Tucker, Steven B. Bleyl, Peter J. Hulick, Ora K. Gordon, Lea Velsher, Jessica Y. J. Gu, Scott M. Weissman, Teresa Kruisselbrink, Christopher Abel, Michele Kettles, Anne Slavotinek, Bryce A. Mendelsohn, Robert C. Green, Swaroop Aradhya, Robert L. Nussbaum

**Affiliations:** 1 Invitae, 1400 16th Street, San Francisco, CA 94103 USA; 2 Stanford University School of Medicine, Stanford, CA USA; 3 Mayo Clinic, Jacksonville, FL USA; 4 Atwal Clinic, Jacksonville, FL USA; 5 PWNHealth, New York, NY USA; 6 Tucker Medical, Singapore, Singapore; 7 Genome Medical, San Francisco, CA USA; 8 NorthShore University HealthSystem, Chicago, IL USA; 9 Providence Research Network, St John Cancer Institute, Los Angeles, CA USA; 10 University of California, Los Angeles, CA USA; 11 Medcan, Toronto, Ontario Canada; 12 Chicago Genetic Consultants, Northbrook, IL USA; 13 Mayo Clinic, Rochester, MN USA; 14 Cooper Clinic, Dallas, TX USA; 15 University of California San Francisco, San Francisco, CA USA; 16 Kaiser Permanente, Oakland, CA USA; 17 Brigham and Women’s Hospital, Boston, MA USA; 18 The Broad Institute, Boston, MA USA; 19 Ariadne Labs, Boston, MA USA; 20 Harvard Medical School, Boston, MA USA; 21 Volunteer Faculty, University of California San Francisco, San Francisco, CA USA

**Keywords:** Cardiovascular disorders, Clinical genetics, Hereditary cancer syndromes, Monogenic disorders, Population screening, Proactive genetic screening

## Abstract

**Background:**

The use of proactive genetic screening for disease prevention and early detection is not yet widespread. Professional practice guidelines from the American College of Medical Genetics and Genomics (ACMG) have encouraged reporting pathogenic variants that confer personal risk for actionable monogenic hereditary disorders, but only as secondary findings from exome or genome sequencing. The Centers for Disease Control and Prevention (CDC) recognizes the potential public health impact of three Tier 1 actionable disorders. Here, we report results of a large multi-center cohort study to determine the yield and potential value of screening healthy individuals for variants associated with a broad range of actionable monogenic disorders, outside the context of secondary findings.

**Methods:**

Eligible adults were offered a proactive genetic screening test by health care providers in a variety of clinical settings. The screening panel based on next-generation sequencing contained up to 147 genes associated with monogenic disorders within cancer, cardiovascular, and other important clinical areas. Sequence and intragenic copy number variants classified as pathogenic, likely pathogenic, pathogenic (low penetrance), or increased risk allele were considered clinically significant and reported. Results were analyzed by clinical area and severity/burden of disease using chi-square tests without Yates’ correction.

**Results:**

Among 10,478 unrelated adults screened, 1619 (15.5%) had results indicating personal risk for an actionable monogenic disorder. In contrast, only 3.1 to 5.2% had clinically reportable variants in genes suggested by the ACMG version 2 secondary findings list to be examined during exome or genome sequencing, and 2% had reportable variants related to CDC Tier 1 conditions. Among patients, 649 (6.2%) were positive for a genotype associated with a disease of high severity/burden, including hereditary cancer syndromes, cardiovascular disorders, or malignant hyperthermia susceptibility.

**Conclusions:**

This is one of the first real-world examples of specialists and primary care providers using genetic screening with a multi-gene panel to identify health risks in their patients. Nearly one in six individuals screened for variants associated with actionable monogenic disorders had clinically significant results. These findings provide a foundation for further studies to assess the role of genetic screening as part of regular medical care.

**Supplementary Information:**

The online version contains supplementary material available at 10.1186/s12916-021-01999-2.

## Background

Screening healthy individuals for genetic risk for monogenic disorders has largely been limited to preconception or prenatal carrier and newborn screening for severe autosomal recessive or X-linked disorders [1]. One exception is the American College of Medical Genetics and Genomics (ACMG) recommendation that clinically significant variants within 59 genes associated with monogenic disorders be analyzed as optional secondary findings during clinically indicated exome or genome sequencing [2, 3]. These 59 genes are primarily associated with adult-onset cancer syndromes and cardiovascular disorders that have established clinical guidelines for further surveillance and preventive therapies. Analyses of large genomic data sets indicate that some asymptomatic individuals carry clinically significant, actionable variants [4, 5].

Emerging evidence supports primary genetic screening for a few hereditary disorders, such as hereditary breast and ovarian cancer syndromes [6, 7]. The Centers for Disease Control and Prevention (CDC) recognizes that hereditary breast and ovarian cancer, familial hypercholesterolemia, and Lynch syndrome have sufficient evidence for intervention that could positively impact public health [8, 9]. Scientists in the UK have made similar recommendations [10, 11], and some countries are characterizing the genomes of large population segments to explore the complexities and precautions to be considered in integrating genomics into health care to provide preventive health information [12]. Finally, some medical institutions are returning genomic information to individuals independent of any existing medical concern, as part of a research study within an integrated health care delivery system [13]. Research studies have shown that most adults offered this type of information consent to receiving it [14, 15].

Recently, the National Academy of Medicine (NAM) published a document on the implementation of genomics-based screening for healthy adults in CDC Tier 1 genes [16]. The statement noted that 1 to 2% of the US population is expected to have a pathogenic variant conferring substantial risk for a serious but preventable disease and, accordingly, acknowledged the opportunity to use screening to identify these otherwise healthy individuals to allow preventive interventions. The statement also called for thoughtfully conducted clinical studies on the implementation of genomic-based screening programs to inform our understanding of their clinical utility.

These developments have created an opportunity to apply genetic screening for actionable disorders to identify personal risk and improve health care. To explore the feasibility and utility of this emerging paradigm, we examined the prevalence of clinically significant variants related to actionable disorders in a large cohort of adults referred from a variety of clinical settings to a commercial laboratory for genetic screening. We anticipated that a notable fraction of individuals who would otherwise not be eligible for genetic testing for hereditary disease would be identified as carrying pathogenic variants that put them at risk for disorders of moderate to high clinical impact.

## Methods

### Gene selection

We reviewed the genes recommended by the ACMG for secondary analyses during exome or genome sequencing [2] and the ClinGen Working Group’s process for selecting genes with clinical actionability [17]. We also examined gene lists used by clinical centers that perform research on reporting personal risk for monogenic disorders [18, 19]. An internal group of geneticists and genetic counselors from Invitae reviewed the clinical actionability of additional genes based on disease severity, penetrance, the availability of published recommendations for medical management, and the strength of gene-disease associations.

The resulting additional genes consisted mainly of those associated with hereditary cancer and cardiovascular conditions beyond those on the ACMG gene list and allowed for a broader spectrum of disease severity. Genes associated with autosomal dominant, autosomal recessive, and X-linked clinical conditions were included. Proactive screening was introduced in 2016 with 124 genes but, after additional curationand strengthening of gene-disease associations, was enlarged to up to 147 genes by the time the study closed in 2020 (Table 1 and Additional file 1: Table S1).

**Table 1:** List of 147 genes for proactive testing

***ACTA2***	***BRCA2***	*CHEK2*	*F9*	*JUP*	***MLH1***	*NTHL1*	*PTCH1*	***SDHC***	*TMEM127*
***ACTC1***	*BRIP1*	***COL3A1***	***FBN1***	*KCNE1*	***MSH2***	***OTC***	***PTEN***	***SDHD***	***TMEM43***
*ACTN2*	*CACNA1C*	*CRYAB*	*FH*	*KCNE2*	*MSH3*	*PALB2*	*RAD51C*	*SGCD*	*TNNC1*
*ACVRL1*	***CACNA1S***	*CSRP3*	*FHL1*	***KCNH2***	***MSH6***	***PCSK9***	*RAD51D*	*SLC40A1*	***TNNI3***
***APC***	*CACNB2*	*DES*	*FLCN*	*KCNJ2*	***MUTYH***	*PDGFRA*	***RB1***	***SMAD3***	***TNNT2***
***APOB***	*CALM1*	*DICER1*	*FLNC*	***KCNQ1***	***MYBPC3***	***PKP2***	*RBM20*	***SMAD4***	***TPM1***
*ATM*	*CALM2*	*DMD*	*GDF2*	*KIT*	***MYH11***	*PLN*	***RET***	*SMARCA4*	*VCL*
***ATP7B***	*CALM3*	***DSC2***	***GLA***	*LAMP2*	***MYH7***	***PMS2***	***RYR1***	*SMARCB1*	***TP53***
*AXIN2*	*CASQ2*	***DSG2***	*GPDIL*	***LDLR***	***MYL2***	*POLD1*	***RYR2***	***STK11***	***TSC1***
*BAG3*	*CAV1*	***DSP***	*GREM1*^*†*^	*LDLRAP1*	***MYL3***	*POLE*	*SERPINA1*	*TCAP*	***TSC2***
*BAP1*	*CAV3*	*EMD*	*HAMP*	***LMNA***	*MYLK*	***PRKAG2***	*SERPINC1*	*TFR2*	***VHL***
*BARD1*	*CDC73*	*ENG*	*HCN4*	*MAX*	*NBN*	*PRKAR1A*	***SCN5A***	*TGFB2*	***WT1***
***BMPR1A***	*CDH1*	*EPCAM*^*†*^	*HFE*	***MEN1***	*NF1*	*PRKG1*	*SDHA*	*TGFB3*	
*BMPR2*	*CDK4*	*F2*^*†*^	*HJV*	*MET*	***NF2***	*PROC*	***SDHAF2***	***TGFBR1***	
***BRCA1***	*CDKN2A*	*F5*^*†*^	*HOXB13*^*†*^	*MITF*^*†*^	*NKX2-5*	*PROS1*	***SDHB***	***TGFBR2***	

Genes shown in bold are the 59 genes prescribed by the ACMG as medically actionable. Genes shown underlined are genes associated with CDC Tier 1 conditions. Genes shown in bold and underlined represent overlap between the two gene lists

^†^Genes that have analytic limitations. *F2*: prothrombin G20210A (c.*97G>A) variant only. *F5*: Factor V Leiden variant only. *GREM1*: promoter region deletion/duplication testing only. *MITF*: c.952G>A, p.Glu318Lys variant only. *HOXB13*: c.251G>A, p.Gly84Glu variant only. *EPCAM*: deletion/duplication testing only

### Patient accrual

Patients were offered a proactive genetic screening test by their health care providers in a variety of domestic and international clinical settings including primary care, executive health, hereditary cancer, and cardiovascular risk clinics. Personal and familial health histories were reviewed, if provided.

Data were collected on individuals 18 years of age or older. Personal or family history of cancer or cardiovascular disease was not an exclusion criterion. However, those who had undergone previous diagnostic genetic testing and were positive for a familial variant associated with a condition represented on the screening panel were excluded from this analysis. Study size was not predetermined but included all patients referred for testing whose samples were returned between January 2016 and May 2020.

### Next-generation sequencing

Each gene was targeted with oligonucleotide baits (Agilent Technologies, Santa Clara, CA; Roche, Pleasanton, CA; IDT, Coralville, IA) to capture exons, the 10 to 20 bases flanking intronic sequences, and noncoding regions of clinical interest. Baits were iteratively balanced to obtain a minimum of 50X and an average of 350X depth-of-sequence read coverage across all targeted areas. Sequencing was performed on HiSeq and NovaSeq instruments (Illumina, San Diego, CA). A suite of bioinformatics methods was used to identify single nucleotide variants, small and large insertions/deletions, exon-level deletions and duplications, and rare structural or mosaic variants [20, 21].

Genomic DNA extracted from patient blood or saliva was processed by next-generation sequencing as described previously [20]. Variants requiring confirmation were confirmed using an orthogonal method, such as PacBio sequencing (Pacific Biosciences, Menlo Park, CA) or exon-focused microarray-based comparative genomic hybridization (Agilent Technologies, Santa Clara, CA) [21]. Clinically reported variants and de-identified clinical information, if provided, were collected for analyses.

### Clinical classification of variants

Sequence and intragenic copy number variants were clinically interpreted using a five-tier system for grading evidence for pathogenicity as implemented in Sherloc [22], a point-based scoring system that expands upon the ACMG/Association for Molecular Pathology variant interpretation guidelines [23]. Variants classified as pathogenic (P), likely pathogenic (LP), pathogenic (low penetrance), or increased risk allele were considered clinically significant and reported. Variants of uncertain significance (VUS) were not reported per professional guidelines [3].

P/LP variants are defined as those that demonstrate the typical penetrance seen in individuals with a disease-associated genotype (i.e., one copy for autosomal dominant conditions or two copies for autosomal recessive conditions). P/LP variants often cause a recognizable Mendelian inheritance pattern of disease, although not all individuals with a disease-associated genotype are affected. Examples include variants in the *BRCA1* and *BRCA2* genes, associated with hereditary breast and ovarian cancer, and variants in the genes associated with Lynch syndrome. Although silent carrier status of a single P/LP variant for a condition that has an autosomal recessive inheritance pattern was reported if present, this type of finding was not considered in the overall yield of clinically significant results.

Pathogenic (low penetrance) variants are found in the same genes where P/LP variants may exist, but their penetrance is measurably lower. Pathogenic (low penetrance) variants may cause a less obvious Mendelian inheritance pattern than P/LP variants, given that fewer individuals with the disease-associated genotype manifest signs of the disorder; nonetheless, a sufficient number of family members will be affected to reveal a Mendelian inheritance pattern. Examples include homozygous *HFE* p.Cys282Tyr or p.His63Asp variants and compound heterozygous p.His63Asp/p.Cys282Tyr variants, associated with hereditary hemochromatosis.

Increased risk alleles are variants that increase risk for a condition that is generally not of sufficient magnitude to reveal a Mendelian inheritance pattern. They are usually identified through association studies comparing the relative risks or odds of disease in individuals with versus without the variants in case-control or cohort studies. Establishing a variant as an increased risk allele based on an association requires the association to meet stringent criteria for statistical significance, effect size, replication, and lack of bias [24]. Increased risk alleles can be more common in a specific population, such as the *APC* p.Ile1307Lys variant, which increases the risk for colon cancer among individuals of Ashkenazi Jewish descent.

### Subdivision of genotypes by impact

Genotypes were subdivided into two categories: “moderate impact” and “high impact.” The level of impact was based on a joint assessment of whether the variants making up the genotype were P/LP, pathogenic (low penetrance), or an increased risk allele and what the severity or burden of the disease associated with that genotype was judged to be. We assigned being heterozygous for certain alleles (i.e., *APC* p.Ile1307Lys, *HOXB13* p.Gly84Glu, or *CHEK2* p.Ile157Thr or p.Ser428Phe), heterozygous for any P/LP allele in *MUTYH*, heterozygous for any P/LP allele in *NBN* except the *NBN* c.657_661delACAAA variant, heterozygous or homozygous for *F2* G20210A or *F5* Leiden, or biallelic for *HFE* and *SERPINA1* pathogenic variants as a moderate impact genotype. All positive results in the other included genes were considered high impact.

### Statistical analysis

Differences in the numbers of patients within specified testing and result categories were compared in 2 × 2 tables using the chi-square test without Yates’ correction and confirmed by the Fisher exact test for 2 × 2 tables. Statistical significance was defined as *p* < 0.05.

## Results

### Demographics

Among 10,478 unrelated adults who underwent proactive screening and genetic analysis, 5367 (51.2%) were between 40 and 59 years old, with an average age of 49.5 years; 6177 (59.0%) were female, and 6274 (59.9%) were self-described as Caucasian (Table 2).

**Table 2:** Cohort demographics

Demographic	Number (%)
**Self-reported ancestry**	
White/Caucasian	6274 (59.9)
Unknown	1608 (15.3)
Asian	604 (5.8)
Multiple ancestries	813 (7.8)
Ashkenazi Jewish	412 (3.9)
Hispanic	246 (2.3)
Black/African American	133 (1.3)
Others	388 (3.7)
**Age in years**	
<20	35 (0.3)
20–29	598 (5.7)
30–39	1965 (18.8)
40–49	2752 (26.3)
50–59	2615 (25.0)
60–69	1746 (16.7)
70–79	682 (6.5)
≥80	85 (0.8)
**Sex**	
Female	6177 (59.0)
Male	4301 (41.0)

Information provided is self-reported ancestry. Age represents the age of an individual at the time of testing

### Positive yield

**Fig. 1:**
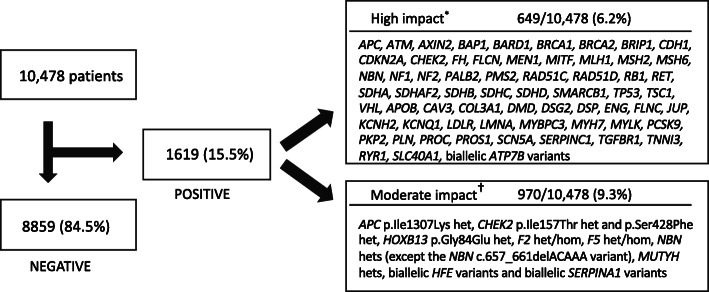
Positive findings, by impact, in a cohort of 10,478 individuals. The asterisk indicates that a genotype was considered high impact when the variants were P/LP and conveyed a substantial risk for disease with substantial burden that requires serious clinical diagnostic and management interventions to prevent or reduce risk from cancer or cardiovascular disease. The dagger indicates that a genotype was considered moderate impact when it contained pathogenic (low penetrance) variants or increased risk alleles (described in the “Methods” section) associated with diseases for which individuals could derive benefit from enhanced surveillance

Using the definitions described in the “Methods” section, screening identified clinically significant results in 1619 (15.5%) of the 10,478 patients (Fig. 1). One hundred thirty-eight individuals harbored multiple variants related to risk for more than one clinical condition, representing 1.3% of this cohort and 8.5% of all positive findings. Overall, 4637 clinically significant sequence variants were reported, including 611 unique variants (Additional file 2: Table S2).

Among all patients, 649 (6.2%) were positive for a genotype with high impact, including P/LP variants associated with a hereditary cancer syndrome, a cardiovascular disorder, or malignant hyperthermia susceptibility. Positive findings of moderate impact were reported in 970 individuals (9.3%), including 450 (4.3%) with a common variant associated with F2- or F5-related thrombophilias, 196 (1.9%) with biallelic variants associated with hereditary hemochromatosis (HH) or alpha-1 antitrypsin deficiency (AATD), and the remaining 324 (3.1%) with genotypes that included other heterozygous pathogenic (low penetrance) or increased risk alleles (Table 4).

### Results by clinical area

Among the 1619 individuals with positive results, 807 (49.8%) had disease-predisposing variants related to a hereditary cancer syndrome. Clinically significant variants were commonly detected in *MUTYH*, *CHEK2*, *APC*, *ATM*, *BRCA1*, *BRCA2*, *MITF*, *HOXB13*, *PMS2*, *PALB2*, *NBN*, *BRIP1*, *MSH6*, *SDHA*, and *BARD1* (Additional file 2: Table S2). Variants in genes associated with colorectal cancer accounted for the highest percentage of reported alleles (including moderate impact heterozygous *MUTYH* variants and *APC* increased risk alleles), followed by variants in genes associated with breast cancer, breast and ovarian cancer, and melanoma (Table 3). Six hundred eight (37.6%) of the 1619 individuals with positive results had variants in genes associated with cardiovascular disorders, with most reportable variants in *F5*, *F2*, *LDLR*, *MYBPC3*, *MYH7*, *APOB*, *SERPINC1*, and *PKP2* (Additional file 2: Table S2). Of the positive cardiovascular disease findings, 158 were high impact variants related to arrhythmias, aortopathies, or cardiomyopathies (88 variants); genes associated with familial hypercholesterolemia (45 variants); and rare forms of hereditary thrombophilia (25 variants). The remaining individuals had moderate impact genotypes in the *F2* (G20210A) and *F5* (Factor V Leiden variant) genes.

**Table 3:** Positive findings grouped by cancer type

Cancer type	Number (%)	Genes with clinically significant variants detected
Gastrointestinal	271 (33.6)	*APC*, *AXIN2*, *CDH1*^†^, *MLH1*, *MSH2*, *MSH6*, *MUTYH*, *PMS2*
Breast	260 (32.2)	*ATM*, *BARD1*, *CHEK2*, *NBN*, *PALB2*, *TP53*^‡^
Breast and ovarian	114 (14.1)	*BRCA1*, *BRCA2*
Melanoma/skin	43 (5.3)	*CDKN2A*, *MITF*, *TGFBR1* (MSSE)
Ovarian	37 (4.6)	*BRIP1*, *RAD51C*, *RAD51D*
Endocrine	33 (4.1)	*MEN1, RET*, *SDHA*, *SDHAF2*, *SDHB*, *SDHC*, *SDHD*
Prostate	27 (3.3)	*HOXB13*
CNS	11 (1.4)	*NF1*, *NF2*^§^, *TSC1*, *VHL*
Others	6 (0.7)	*BAP1*, *RB1*, *SMARCB1*
Renal	5 (0.6)	*FH*, *FLCN*

*CNS* central nervous system

Indications of higher risk from screening genes associated with hereditary cancer syndromes were most commonly related to gastrointestinal, breast, ovarian, and skin cancers. The genetic changes recorded here for each gene represent a heterozygous finding associated with an autosomal dominant condition, or a single heterozygous variant in the *MUTYH* gene. Certain genetic changes in the *TGFBR1* gene can cause multiple self-healing squamous epithelioma (MSSE), which was classified as a hereditary cancer risk

^†^*CDH1* is also associated with breast cancer risk

^‡^*TP53* is associated with Li-Fraumeni syndrome, which is associated with multiple cancer types

^§^*NF2* is associated with non-malignant nervous system tumors

### Yield from ACMG and CDC Tier 1 prescribed genes

Restricting results to only the 59 genes recommended by the ACMG for secondary analysis during exome or genome sequencing (version 2) [3] demonstrated a positive yield of 5.2% (Table 4) when including all high impact P/LP variants and all moderate impact increased risk alleles and genotypes regardless of inheritance pattern. Monoallelic changes that result in silent carrier status without disease were not included in any yield calculation. When increased risk alleles and genotypes with moderate impact were excluded, the positive yield decreased to 3.1%. Similarly, when positive results were limited to the CDC Tier 1 genes (*BRCA1*, *BRCA2*, *MLH1*, *MSH2*, *MSH6*, *EPCAM*, *PMS2*, *APOB*, *LDLR*, and *PCSK9*), the yield was 2% (Table 4).

**Table 4:** Stratification of findings by genes or variants

All findings	Number (%)
Individuals with reportable findings	1619 (15.5)
Yield after excluding biallelic *HFE* and *SERPINA1* findings	1423 (13.6)
Yield after excluding *F2* and *F5* findings and genes in the row above	973 (9.3)
Yield after excluding *MUTYH* heterozygotes and genes in rows above	829 (7.9)
Yield after excluding *CHEK2* p.Ile157Thr and p.Ser428Phe heterozygotes, *NBN* heterozygotes (except for the *NBN* c.657_661delACAAA variant), and genes in rows above	746 (7.1)
Yield after excluding increased risk alleles (*APC* p.I1307K, *HOXB13* p.Gly84Glu) and genes or variants in rows above	649 (6.2)
**Findings in 59 genes prescribed by the ACMG**	
Individuals with reportable findings in the 59 ACMG genes	542 (5.2)
Yield after excluding *MUTYH* heterozygotes and increased risk alleles	326 (3.1)
**Findings in CDC Tier 1 genes**	
All reportable findings in genes associated with HBOC, FH, and Lynch syndrome	205 (2.0)
HBOC findings only	114 (1.1)
Lynch syndrome findings only	51 (0.5)
FH findings only	40 (0.4)

*ACMG* American College of Medical Genetics and Genomics, *CDC* Centers for Disease Control and Prevention, *HBOC* hereditary breast and ovarian cancer, *FH* familial hypercholesterolemia

Stratification of results in the 59 genes prescribed by the ACMG is done by incrementally removing specific categories of gene groups (shown in successive rows) that are excluded per ACMG secondary findings guidelines. Similarly, stratification of results from CDC Tier 1 genes is shown based on different disease categories: HBOC syndrome (*BRCA1* and *BRCA2*), Lynch syndrome (*EPCAM*, *MLH1*, *MSH2*, *MSH6*, and *PMS2*), and FH (*APOB* gain-of-function variants, *LDLR*, and *PCSK9*)

Among all 10,478 individuals, 1077 (10.3%) had a clinically significant variant in a gene that was not included in the 59 ACMG genes: 3.7% in a cancer-related gene, 4.7% in a gene associated with a cardiovascular disorder (including the thrombophilias), and 1.9% with biallelic variants associated with HH or AATD.

### Implications of provided health information

We received voluntary health histories from 6710 (64.0%) of the 10,478 individuals. Among the 6710 returning health histories, 96 (1.4%) reported an absence of family health information due to adoption, 2801 (41.7%) indicated no relevant personal or family history, 242 (3.6%) disclosed a personal history of cancer, 2409 (35.9%) reported a family history of cancer, and 411 (6.1%) reported both a personal and family history of cancer. Similarly, 328 (4.9%) had a personal or family history of cardiovascular disorders, and 423 (6.3%) had a personal or family history of both cancer and cardiovascular conditions.

Comparing the positive yield of hereditary cancer syndrome tests among individuals with different cancer histories, 4.3% of individuals (120/2801) without a personal or family history had a P/LP finding in one of the hereditary cancer syndrome genes (excluding the *APC* increased risk allele and *MUTYH* heterozygotes). This was significantly lower than the 9.5% (62/653, *χ*^2^ = 28.8, *p*<< 10^−5^, 1 d.f.) positivity rate in individuals reporting a *personal* history of cancer (with or without a family history) and significantly lower than the 7.1% (171/2409, *χ*^2^ = 19.45, *p*<< 10^−5^, 1 d.f.) positivity rate in individuals with only a *family* history of cancer. There was no significant difference in the positivity rate among individuals with personal history alone (8.7%), family history alone (7.1%), or both personal and family history (10%) (*χ*^2^ = 4.56, *p*=0.10, 2 d.f.).

## Discussion

Recent discussion has centered around the potential of proactive genetic screening to determine personal risk for actionable monogenic disorders and improve public health outcomes [16, 25, 26]. The overall positive yield of 15.5% in our cohort of 10,478 individuals represents an early example of the expected yield of proactive screening implemented in a variety of practice settings. A yield of 1.5 to 6% has been reported in secondary findings for the 59 genes that the ACMG suggests be analyzed during whole exome or genome sequencing [5, 15, 27], and large databases containing genomic information from healthy individuals have demonstrated a comparable yield [4, 28, 29]. We found a similar yield of 3.1% in the 59 genes when we applied the narrower criteria for reporting variants as described by the ACMG [2, 3]. These results are clinically meaningful because variants in these genes confer a high risk of serious disease compared with the risk in the general population. Identifying this risk facilitates the application of precision preventive medical interventions, including appropriate screening protocols to either prevent disease or detect it early to reduce morbidity and mortality [6].

The clinical yield in the 59 ACMG genes increased from 3.1 to 5.2% when the *APC* p.Ile1307Lys increased risk allele and *MUTYH* heterozygotes were included. However, debate continues about whether variants in genes that may confer only low risk for disease should be reported [30, 31]. Although these variants confer lower risk than variants in other tested genes, the cancer risk they confer is still higher than the risk in the general population and can trigger changes in risk management. For example, for individuals of Ashkenazi Jewish ancestry, the *APC* p.I1307K increased risk allele indicates an elevated risk of colorectal cancer that warrants modifications to clinical management [31, 32]. Similarly, all individuals with a single, heterozygous P/LP variant in *MUTYH* have an estimated twofold increased risk of colorectal cancer [30, 33], and current guidelines indicate that carriers who have a first-degree relative with colorectal cancer should consider more frequent colonoscopy screenings [34]. Such cases represent additional opportunities for precision preventive medical interventions that could otherwise be missed without genetic screening.

When using genetic screening to assess personal risk, reduced specificity in classifying variants can lead to false-positive clinical results, particularly for rare disorders [35]. To increase test specificity and positive predictive values [33], only well-established pathogenic variants or those predicted with high confidence to be pathogenic were reported. In contrast, VUS were not reported, per professional guidelines and the NAM’s proposal for genomics-based screening [16, 25, 26]. During this study, however, a few variant interpretations were changed, resulting in amended reports. Some variants initially documented as clinically significant were downgraded to VUS, while some VUS (which had not been reported) were reclassified as clinically significant as a result of new evidence. Reclassification of VUS to clinically significant is a particular challenge because the individuals and their providers were not aware of these variants until they received amended reports, giving the false impression that clinically relevant variants had been missed during initial testing. Nonetheless, given that a duty to reinterpret variants and inform patients and their providers of clinically impactful changes has been proposed [36], amended reports were provided. This highlights the importance of disclosing during pre-test counseling that a report could be amended if new information becomes available.

Our findings also show that individuals with personal or family history of cancer or cardiovascular disorders who may not meet guidelines-based criteria for diagnostic genetic testing often seek proactive genetic screening. Such individuals have historically had few options for obtaining physician-directed clinical genetic testing. Recent studies show that a substantial proportion of individuals with cancer who do not meet established clinical criteria for testing have a rate of clinically significant findings comparable to that of individuals who do meet criteria [37, 38]. A similar pattern was observed in a recent study reporting secondary findings in the 59 ACMG-recommended genes [15]. That study demonstrated a significantly greater yield of P/LP variants in hereditary cancer syndrome genes in individuals with a personal or family history of cancer than in individuals with no such history; however, no statistical differences were found in the yield of medically actionable variants among individuals with a personal history of cancer, a family history of cancer, or both. In this study, although 1 in 23 healthy individuals with no personal or family history of cancer received a medically actionable result for a hereditary cancer syndrome gene, screening was even more informative for individuals who had a personal or family history but may not have met genetic testing criteria within existing standards, for whom the positivity rate was about two- to threefold higher [39].

Another potential benefit of genetic screening is the ability to find individuals with undiagnosed Mendelian disease presenting in primary care practices. This has been demonstrated by the application of a phenotype risk score (developed by mapping clinical features of Mendelian diseases into phenotypes derived from electronic health records) in which phenotype-genotype associations were used to identify patients with five Mendelian disorders that had previously been undiagnosed or diagnosed incorrectly in a primary care setting [40]. One may conclude that many individuals with significant risk for, or already manifesting, actionable hereditary disorders are likely escaping diagnosis [41]. In fact, the genes responsible for three of the five disorders that went undiagnosed but were flagged by the phenotype risk score (i.e., Marfan syndrome, hereditary hemochromatosis, and Li-Fraumeni syndrome) were part of the proactive screening panel. Proactive genetic screening could identify more at-risk individuals, especially as technologies make it easier to screen more broadly and variant interpretation capabilities continue to improve.

Complex and interrelated challenges will impact the use and utility of genetic screening for assessing personal risk for actionable disorders. First, inadequate understanding of the penetrance associated with these disorders, differences in risk based on ethnicity or lifestyle, and the spectra of variants in diverse populations may limit the current ability of proactive genetic screening to precisely determine risks [42]. As a result of differences in penetrance, the probability that a clinically significant finding will accurately predict whether someone eventually develops that condition may vary between symptomatic individuals and healthy individuals without a personal or family history. Although disease risk may be lower in the latter group, it is still expected to be higher than in the general population. This was the premise behind the ACMG secondary findings guidelines and the NAM recommendations. In fact, we have seen in this clinical cohort and other studies [15, 40] that a small number of individuals who are considered healthy and undergo recommended clinical follow-up due to a genetic variant identified through proactive genetic screening are found to be affected with a subclinical or atypical Mendelian phenotype. It is therefore important that positive reports clearly encourage further clinical evaluation, including increased surveillance and ongoing monitoring, but that they not be used in isolation to justify irreversible clinical actions. As longitudinal studies worldwide provide better estimates of penetrance for many disorders, the precision of the predictive value of population-level screening will increase. These issues as well as additional challenges associated with implementing DNA-based population screening programs will require further consideration and are beginning to be discussed broadly [43, 44].

Limited genetics expertise in adult primary care poses another challenge to the broader use of proactive genetic screening. The National Society of Genetic Counselors, the ACMG, and other professional societies are developing professional practice guidelines for personal genetic risk assessment [45, 46] and novel service delivery models for pre- and post-test genetic counseling [47, 48]. Because of the persistent shortage of genetics professionals, consideration and research must continue to be devoted to developing these models as well as scalable mechanisms for communicating genomic test results to more individuals and coordinating appropriate medical follow-up.

## Conclusions

This large multi-center cohort study is one of the first real-world examples of specialists and primary care providers using genetic screening with a targeted multi-gene panel to identify health risks in their patients. Nearly one in six individuals screened for variants associated with actionable monogenic disorders had clinically significant results. Many of these results were in genes beyond the 59 recommended for secondary reporting by the ACMG (version 2) or the 10 recognized as Tier 1 genes (associated with three Tier 1 conditions) by the CDC.

Our results are a first step toward gathering the data needed to address the medical, ethical, and economic implications of proactive genetic screening. These data also provide a foundation for further studies to assess the role of genetic screening, as part of regular medical care, in reducing morbidity and mortality from actionable genetic disorders and to determine its clinical utility and cost-effectiveness.

## Supplementary Information

**Additional file 1: Table S1.** Details of genes included in proactive screening.

**Additional file 2: Table S2.** Clinically significant variants detected in this patient cohort.

## Data Availability

Most of the datasets generated or analyzed during the current study are shared in this published article. Other data that support the findings of this study are available from the corresponding author upon reasonable request. In addition, all variants are shared with the public database ClinVar for patients who provide consent. ClinVar can be accessed at https://www.ncbi.nlm.nih.gov/clinvar/.

## Abbreviations

AATD: Alpha-1 antitrypsin deficiency
ACMG: American College of Medical Genetics and Genomics
CDC: Centers for Disease Control and Prevention
HH: Hereditary hemochromatosis
LP: Likely pathogenic
NAM: National Academy of Medicine
P: Pathogenic
