# Correction to: Physician-directed genetic screening to evaluate personal risk for medically actionable disorders: a large multi-center cohort study

**DOI:** 10.1186/s12916-021-02141-y

**Published:** 2021-11-03

**Authors:** Eden V. Haverfield, Edward D. Esplin, Sienna J. Aguilar, Kathryn E. Hatchell, Kelly E. Ormond, Andrea Hanson-Kahn, Paldeep S. Atwal, Sarah Macklin-Mantia, Stephanie Hines, Caron W.-M. Sak, Steven Tucker, Steven B. Bleyl, Peter J. Hulick, Ora K. Gordon, Lea Velsher, Jessica Y. J. Gu, Scott M. Weissman, Teresa Kruisselbrink, Christopher Abel, Michele Kettles, Anne Slavotinek, Bryce A. Mendelsohn, Robert C. Green, Swaroop Aradhya, Robert L. Nussbaum

**Affiliations:** 1 Invitae, 1400 16th Street, San Francisco, CA 94103 USA; 2 Stanford University School of Medicine, Stanford, CA USA; 3 Mayo Clinic, Jacksonville, FL USA; 4 Atwal Clinic, Palm Beach, FL USA; 5 PWNHealth, New York, NY USA; 6 Tucker Medical, Singapore, Singapore; 7 Genome Medical, San Francisco, CA USA; 8 NorthShore University Health System, Chicago, IL USA; 9 Providence Research Network, St John Cancer Institute, Los Angeles, CA USA; 10 University of California, Los Angeles, CA USA; 11 Medcan, Toronto, Ontario Canada; 12 Chicago Genetic Consultants, Northbrook, IL USA; 13 Mayo Clinic, Rochester, MN USA; 14 Cooper Clinic, Dallas, TX USA; 15 University of California San Francisco, San Francisco, CA USA; 16 Kaiser Permanente, Oakland, CA USA; 17 Brigham and Women’s Hospital, Boston, MA USA; 18 The Broad Institute, Boston, MA USA; 19 Ariadne Labs, Boston, MA USA; 20 Harvard Medical School, Boston, MA USA; 21 Volunteer Faculty, University of California San Francisco, San Francisco, CA USA

**Correction to: BMC Med 19, 199 (2021)**

**https://doi.org/10.1186/s12916-021-01999-2**

The original article [1] contained numerous typos which were mistakenly carried forward by the team that managed production of this article.

These errors have since been corrected.
