# Supplementary material for: Physician-directed genetic screening to evaluate personal risk for medically actionable disorders: a large multi-center cohort study
Source: BMC Med. 2021 Aug 18;19:199. doi: 10.1186/s12916-021-01999-2 (PMC8371767; doi:10.1186/s12916-021-01999-2)
Supplement: Supplementary file 1 — Additional file 1: Table S1. Details of genes included in proactive screening. [file 12916_2021_1999_MOESM1_ESM.pdf]

Table S1. Details of genes included in proactive screening.

Gene	Conditions*	MIM	Inheritance Patterns
ACTA2	Thoracic aortic aneurysms and dissections	102620	AD
ACTC1	Atrial septal defect<br>Dilated cardiomyopathy<br>Hypertrophic cardiomyopathy<br>Left ventricular noncompaction cardiomyopathy	102540	AD
ACTN2	Dilated cardiomyopathy<br>Hypertrophic cardiomyopathy	102573	AD
ACVRL1	Hereditary hemorrhagic telangiectasia<br>Pulmonary hypertension	601284	AD
APC	Familial adenomatous polyposis (FAP) and attenuated FAP	611731	AD
APOB	Familial hypercholesterolemia<br>Familial hypobetalipoproteinemia	107730	AD, AR
ATM	*ATM*-related cancers	607585	AD, AR
ATP7B	Wilson disease	606882	AR
AXIN2	Oligodontia-colorectal cancer syndrome	604025	AD
BAG3	Dilated cardiomyopathy<br>Myofibrillar myopathy	603883	AD
BAP1	*BAP1* tumor predisposition syndrome	603089	AD
BARD1	*BARD1*-related cancers	601593	AD
BMPR1A	Juvenile polyposis syndrome	601299	AD
BMPR2	Pulmonary arterial hypertension	600799	AD
BRCA1	Hereditary breast and ovarian cancer syndrome<br>Fanconi anemia	113705	AD, AR
BRCA2	Hereditary breast and ovarian cancer syndrome<br>Fanconi anemia	600185	AD, AR
BRIP1	*BRIP1*-related cancers<br>Fanconi anemia	605882	AD, AR
CACNA1C	Brugada syndrome<br>Short QT syndrome<br>Timothy syndrome	114205	AD
CACNA1S	Hypokalemic periodic paralysis<br>Malignant hyperthermia susceptibility	114208	AD
CACNB2	Brugada syndrome<br>Short QT syndrome	600003	AD
CALM1	Catecholaminergic polymorphic ventricular tachycardia<br>Long QT syndrome	114180	AD
CALM2	Catecholaminergic polymorphic ventricular tachycardia<br>Long QT syndrome	114182	AD
CALM3	Catecholaminergic polymorphic ventricular tachycardia<br>Long QT syndrome	114183	AD
CASQ2	Catecholaminergic polymorphic ventricular tachycardia	114251	AR
CAV1	Pulmonary arterial hypertension	601047	AD
CAV3	*CAV3*-related neuromuscular conditions<br>Hypertrophic cardiomyopathy<br>Long QT syndrome	601253	AD

CDC73	Hyperparathyroidism-jaw tumor syndrome<br>Familial isolated hyperparathyroidism<br>Parathyroid carcinoma	607393	AD
CDH1	Hereditary diffuse gastric cancer syndrome	192090	AD
CDK4	Hereditary cutaneous melanoma	123829	AD
CDKN2A	Hereditary melanoma-pancreatic cancer syndrome	600160	AD
CHEK2	*CHEK2* -related cancers	604373	AD
COL3A1	Ehlers-Danlos syndrome, vascular type	120180	AD
CRYAB	Dilated cardiomyopathy<br>Myofibrillar myopathy	123590	AD, AR
CSRP3	Dilated cardiomyopathy<br>Hypertrophic cardiomyopathy	600824	AD
DES	Dilated cardiomyopathy<br>Myofibrillar myopathy	125660	AD, AR
DICER1	*DICER1* -related pleuropulmonary blastoma familial tumor predisposition syndrome	606241	AD
DMD	Becker muscular dystrophy<br>Dilated cardiomyopathy<br>Duchenne muscular dystrophy	300377	X-linked
DSC2	Arrhythmogenic right ventricular cardiomyopathy	125645	AD, AR
DSG2	Arrhythmogenic right ventricular cardiomyopathy<br>Dilated cardiomyopathy	125671	AD, AR
DSP	Arrhythmogenic right ventricular cardiomyopathy<br>Dilated cardiomyopathy	125647	AD, AR
EMD	Emery-Dreifuss muscular dystrophy	300384	X-linked
ENG	Hereditary hemorrhagic telangiectasia	131195	AD
EPCAM	Lynch syndrome	185535	AD
F2	Hereditary thrombophilia	176930	AD, AR
F5	Hereditary thrombophilia	612309	AD, AR
F9	Hemophilia<br>Hereditary thrombophilia	300746	X-linked
FBN1	*FBN1* -related conditions	134797	AD
FH	Hereditary leiomyomatosis and renal cell cancer<br>Fumarase deficiency	136850	AD, AR
FHL1	Emery-Dreifuss muscular dystrophy<br>Hypertrophic cardiomyopathy<br>Reducing body myopathy	300163	X-Linked
FLCN	Birt-Hogg-Dubé syndrome	607273	AD
FLNC	Dilated cardiomyopathy<br>Distal myopathy<br>Hypertrophic cardiomyopathy<br>Myofibrillar myopathy<br>Restrictive cardiomyopathy	102565	AD
GDF2	Hereditary hemorrhagic telangiectasia	605120	AD
GLA	Fabry disease	300644	X-linked
GPD1L	Brugada syndrome	611778	AD
GREM1	Hereditary mixed polyposis syndrome	603054	AD
HAMP	Hereditary hemochromatosis	606464	AR

HCN4	Brugada syndrome<br>Left ventricular noncompaction cardiomyopathy<br>Sinus node dysfunction or bradycardia	605206	AD
HFE	Hereditary hemochromatosis	613609	AR
HJV	Hereditary hemochromatosis	608374	AR
HOXB13	Prostate cancer	604607	AD
JUP	Arrhythmogenic right ventricular cardiomyopathy<br>Naxos disease	173325	AD, AR
KCNE1	Long QT syndrome<br>Jervell and Lange-Nielsen syndrome	176261	AD, AR
KCNE2	Long QT syndrome	603796	AD
KCNH2	Brugada syndrome<br>Long QT syndrome<br>Short QT syndrome	152427	AD
KCNJ2	Andersen-Tawil syndrome<br>Catecholaminergic polymorphic ventricular tachycardia<br>Short QT syndrome	600681	AD
KCNQ1	Atrial fibrillation<br>Long QT syndrome<br>Short QT syndrome<br>Jervell and Lange-Nielsen syndrome	607542	AD, AR
KIT	Gastrointestinal stromal tumors	164920	AD
LAMP2	Danon disease	309060	X-linked
LDLR	Familial hypercholesterolemia	606945	AD, AR
LDLRAP1	Familial hypercholesterolemia	605747	AR
LMNA	Congenital muscular dystrophy<br>Dilated cardiomyopathy<br>Emery-Dreifuss muscular dystrophy<br>Limb-girdle muscular dystrophy	150330	AD, AR
MAX	Hereditary paraganglioma-pheochromocytoma syndrome	154950	AD
MEN1	Multiple endocrine neoplasia type 1	613733	AD
MET	Hereditary papillary renal cell carcinoma	164860	AD
MITF	Hereditary cutaneous malignant melanoma	156845	AD
MLH1	Lynch syndrome<br>Constitutional mismatch repair deficiency syndrome	120436	AD, AR
MSH2	Lynch syndrome<br>Constitutional mismatch repair deficiency syndrome	609309	AD, AR
MSH3	*MSH3*-associated polyposis	600887	AR
MSH6	Lynch syndrome<br>Constitutional mismatch repair deficiency syndrome	600678	AD, AR
MUTYH	*MUTYH*-associated polyposis	604933	AR
MYBPC3	Dilated cardiomyopathy<br>Hypertrophic cardiomyopathy<br>Left ventricular noncompaction cardiomyopathy	600958	AD
MYH11	Thoracic aortic aneurysms and dissections	160745	AD

MYH7	Dilated cardiomyopathy<br>Hypertrophic cardiomyopathy<br>Laing distal myopathy<br>Left ventricular noncompaction cardiomyopathy<br>Myosin storage myopathy	160760	AD, AR
MYL2	Hypertrophic cardiomyopathy	160781	AD
MYL3	Hypertrophic cardiomyopathy<br>Restrictive cardiomyopathy	160790	AD, AR
MYLK	Thoracic aortic aneurysms and dissections	600922	AD
NBN	*NBN*-related cancers<br>Nijmegen breakage syndrome	602667	AD, AR
NF1	Neurofibromatosis type 1<br>Neurofibromatosis-Noonan syndrome<br>Watson syndrome	613113	AD
NF2	Neurofibromatosis type 2	607379	AD
NKX2-5	*NKX2-5* related cardiovascular conditions	600584	AD
NTHL1	*NTHL1*-associated polyposis	602656	AR
OTC	Ornithine transcarbamylase deficiency	300461	X-linked
PALB2	*PALB2*-related cancers<br>Fanconi anemia	610355	AD, AR
PCSK9	Familial hypercholesterolemia	607786	AD
PDGFRA	Gastrointestinal stromal tumors	173490	AD
PKP2	Arrhythmogenic right ventricular cardiomyopathy<br>Brugada syndrome<br>Dilated cardiomyopathy	602861	AD
PLN	Arrhythmogenic right ventricular cardiomyopathy<br>Dilated cardiomyopathy<br>Hypertrophic cardiomyopathy	172405	AD
PMS2	Lynch syndrome<br>Constitutional mismatch repair deficiency syndrome	600259	AD, AR
POLD1	*POLD1*-related cancers<br>Mandibular hypoplasia, deafness, progeroid features, and lipodystrophy	174761	AD
POLE	*POLE* -related cancers<br>Facial dysmorphism, immunodeficiency, livedo, and short stature (FILS) syndrome	174762	AD, AR
PRKAG2	Hypertrophic cardiomyopathy<br>Wolff-Parkinson-White syndrome	602743	AD
PRKAR1A	Carney complex	1188830	AD
PRKG1	Thoracic aortic aneurysms and dissections	176894	AD
PROC	Hereditary thrombophilia	612283	AD, AR
PROS1	Hereditary thrombophilia	176880	AD, AR
PTCH1	Basal cell nevus syndrome	601309	AD
PTEN	*PTEN* hamartoma tumor syndrome	601728	AD
RAD51C	*RAD51C*-related cancers	602774	AD
RAD51D	*RAD51D*-related cancers	602954	AD
RB1	Retinoblastoma	614041	AD
RBM20	Dilated cardiomyopathy	613171	AD

RET	Multiple endocrine neoplasia type 2	164761	AD
RYR1	Central core disease<br>Centronuclear myopathy<br>Malignant hyperthermia susceptibility	180901	AD, AR
RYR2	Arrhythmogenic right ventricular cardiomyopathy<br>Catecholaminergic polymorphic ventricular tachycardia<br>Left ventricular noncompaction cardiomyopathy	180902	AD
SCN5A	Atrial fibrillation<br>Brugada syndrome<br>Dilated cardiomyopathy<br>Long QT syndrome	600163	AD
SERPINA1	Alpha-1 antitrypsin deficiency	107400	AR
SERPINC1	Hereditary thrombophilia	107300	AD, AR
SDHA	Hereditary paraganglioma-pheochromocytoma syndrome<br>Gastrointestinal stromal tumors<br>Mitochondrial complex II deficiency	600857	AD, AR
SDHAF2	Hereditary paraganglioma-pheochromocytoma syndrome	613019	AD
SDHB	Hereditary paraganglioma-pheochromocytoma syndrome<br>Gastrointestinal stromal tumors<br>Renal cancer<br>Mitochondrial complex II deficiency	185470	AD, AR
SDHC	Hereditary paraganglioma-pheochromocytoma syndrome<br>Gastrointestinal stromal tumors<br>Renal cancer	602413	AD
SDHD	Hereditary paraganglioma-pheochromocytoma syndrome<br>Gastrointestinal stromal tumors	602690	AD
SGCD	Limb-girdle muscular dystrophy	601411	AR
SLC40A1	Hereditary hemochromatosis	604653	AR
SMAD3	Loeys-Dietz syndrome<br>Thoracic aortic aneurysms and dissections	603109	AD
SMAD4	Hereditary hemorrhagic telangiectasia<br>Juvenile polyposis syndrome	600993	AD
SMARCA4	*SMARCA4*-related cancers	603254	AD
SMARCB1	Rhabdoid tumor predisposition syndrome<br>Schwannomatosis	601607	AD
STK11	Peutz-Jeghers syndrome	602216	AD
TCAP	Dilated cardiomyopathy<br>Limb-girdle muscular dystrophy	604488	AD, AR
TFR2	Hereditary hemochromatosis	604720	AR
TGFB2	Loeys-Dietz syndrome	190220	AD
TGFB3	Arrhythmogenic right ventricular cardiomyopathy<br>Loeys-Dietz syndrome	190230	AD
TGFBR1	Loeys-Dietz syndrome<br>Thoracic aortic aneurysms and dissections<br>Multiple self-healing squamous epithelioma	190181	AD
TGFBR2	Loeys-Dietz syndrome<br>Thoracic aortic aneurysms and dissections	190182	AD
TMEM127	Hereditary paraganglioma-pheochromocytoma syndrome	613403	AD

TMEM43	Arrhythmogenic right ventricular cardiomyopathy	612048	AD
TNNC1	Dilated cardiomyopathy<br>Hypertrophic cardiomyopathy	191040	AD
TNNI3	Dilated cardiomyopathy<br>Hypertrophic cardiomyopathy<br>Restrictive cardiomyopathy	191044	AD
TNNT2	Dilated cardiomyopathy<br>Hypertrophic cardiomyopathy<br>Left ventricular noncompaction cardiomyopathy<br>Restrictive cardiomyopathy	191045	AD
TPM1	Dilated cardiomyopathy<br>Hypertrophic cardiomyopathy<br>Left ventricular noncompaction cardiomyopathy	191010	AD
VCL	Dilated cardiomyopathy	193065	AD
TP53	Li-Fraumeni syndrome	191170	AD
TSC1	Tuberous sclerosis complex	605284	AD
TSC2	Tuberous sclerosis complex	191092	AD
VHL	Von Hippel-Lindau syndrome<br>Familial erythrocytosis, type 2	608537	AD, AR
WT1	*WT1*-related Wilms tumor	607102	AD

AD = autosomal dominant; AR = autosomal recessive.*Genes listed in this table may also have additional reported clinical associations outside of the conditions listed. Additional information about gene-condition associations can be found at http://www.omim.org
