# Supplementary material for: Physician-directed genetic screening to evaluate personal risk for medically actionable disorders: a large multi-center cohort study
Source: BMC Med. 2021 Aug 18;19:199. doi: 10.1186/s12916-021-01999-2 (PMC8371767; doi:10.1186/s12916-021-01999-2)
Supplement: Supplementary file 2 — Additional file 2: Table S2. Clinically significant variants detected in this patient cohort. [file 12916_2021_1999_MOESM2_ESM.pdf]

Table S2. Clinically significant variants detected in this patient cohort. For further details related to the classification of each variant, see https://www.ncbi.nlm.nih.gov/clinvar/

Gene	Variant (HGVS)	Effect	Variant Type	Interpretation
APC	NM_000038.5:c.4733_4734del	p.Cys1578Tyrfs	indel	Pathogenic
APC	NM_000038.5:c.1415dup	p.Gln473Thrfs	indel	Pathogenic
APC	NM_000038.5:c.3920T>A	p.Ile1307Lys	substitution	Increased Risk Allele
APOB	NM_000384.2:c.6543del	p.Phe2181Leufs	indel	Pathogenic
APOB	NM_000384.2:c.671del	p.Pro224Hisfs	indel	Pathogenic
APOB	NM_000384.2:c.7537C>T	p.Arg2513*	substitution	Pathogenic
APOB	NM_000384.2:c.10580G>A	p.Arg3527Gln	substitution	Pathogenic
APOB	NM_000384.2:c.10579C>T	p.Arg3527Trp	substitution	Pathogenic
APOB	NM_000384.2:c.11764C>T	p.Gln3922*	substitution	Pathogenic
APOB	NM_000384.2:c.11789-1G>C	Splice site	substitution	Likely Pathogenic
ATM	NM_000051.3:c.5675-?_5762+?del	Deletion (Exon 38)	cnv	Pathogenic
ATM	NM_000051.3:c.8851-?_*3591+?del	Deletion (Exons 62-63)	cnv	Pathogenic
ATM	NM_000051.3:c.7517_7520del	p.Arg2506Thrfs	indel	Pathogenic
ATM	NM_000051.3:c.7638_7646del	p.Arg2547_Ser2549del	indel	Pathogenic
ATM	NM_000051.3:c.4683_4689del	p.Asp1563Phefs	indel	Pathogenic
ATM	NM_000051.3:c.7010_7011del	p.Cys2337Serfs	indel	Pathogenic
ATM	NM_000051.3:c.8823_8824del	p.Gln2942Glyfs	indel	Pathogenic
ATM	NM_000051.3:c.1027_1030del	p.Glu343Ilefs	indel	Pathogenic
ATM	NM_000051.3:c.1564_1565del	p.Glu522Ilefs	indel	Pathogenic
ATM	NM_000051.3:c.7886_7890del	p.Ile2629Serfs	indel	Pathogenic
ATM	NM_000051.3:c.6228del	p.Leu2077Phefs	indel	Pathogenic
ATM	NM_000051.3:c.7669_7670del	p.Leu2557Valfs	indel	Pathogenic
ATM	NM_000051.3:c.2284_2285del	p.Leu762Valfs	indel	Pathogenic
ATM	NM_000051.3:c.8432del	p.Lys2811Serfs	indel	Pathogenic
ATM	NM_000051.3:c.5894_5900dup	p.Met1967Ilefs	indel	Pathogenic
ATM	NM_000051.3:c.2838del	p.Met946Ilefs	indel	Pathogenic
ATM	NM_000051.3:c.5631_5635delinsA	p.Phe1877Leufs	indel	Pathogenic
ATM	NM_000051.3:c.1597_1600dup	p.Pro534Glnfs	indel	Pathogenic
ATM	NM_000051.3:c.6997dup	p.Thr2333Asnfs	indel	Pathogenic
ATM	NM_000051.3:c.1355del	p.Thr452Asnfs	indel	Pathogenic
ATM	NM_000051.3:c.7542_7543delTA	p.Tyr2514*	indel	Pathogenic
ATM	NM_000051.3:c.3802del	p.Val1268*	indel	Pathogenic
ATM	NM_000051.3:c.381delA	p.Val128*	indel	Pathogenic
ATM	NM_000051.3:c.4804_4805del	p.Val1602Leufs	indel	Pathogenic
ATM	NM_000051.3:c.1997del	p.Val666Glyfs	indel	Pathogenic
ATM	NM_000051.3:c.5177+5G>A	Intronic	substitution	Likely Pathogenic
ATM	NM_000051.3:c.5763-1050A>G	Intronic	substitution	Pathogenic
ATM	NM_000051.3:c.5825C>T	p.Ala1942Val	substitution	Pathogenic
ATM	NM_000051.3:c.4396C>T	p.Arg1466*	substitution	Pathogenic
ATM	NM_000051.3:c.5623C>T	p.Arg1875*	substitution	Pathogenic
ATM	NM_000051.3:c.67C>T	p.Arg23*	substitution	Pathogenic
ATM	NM_000051.3:c.8977C>T	p.Arg2993*	substitution	Pathogenic
ATM	NM_000051.3:c.103C>T	p.Arg35*	substitution	Pathogenic
ATM	NM_000051.3:c.5515C>T	p.Gln1839*	substitution	Pathogenic
ATM	NM_000051.3:c.2341C>T	p.Gln781*	substitution	Pathogenic
ATM	NM_000051.3:c.5932G>T	p.Glu1978*	substitution	Pathogenic
ATM	NM_000051.3:c.6115G>A	p.Glu2039Lys	substitution	Likely Pathogenic
ATM	NM_000051.3:c.841G>T	p.Glu281*	substitution	Pathogenic
ATM	NM_000051.3:c.967A>G	p.Ile323Val	substitution	Pathogenic
ATM	NM_000051.3:c.8549T>A	p.Leu2850*	substitution	Pathogenic
ATM	NM_000051.3:c.2849T>G	p.Leu950Arg	substitution	Likely Pathogenic
ATM	NM_000051.3:c.5890A>T	p.Lys1964*	substitution	Pathogenic
ATM	NM_000051.3:c.8266A>T	p.Lys2756*	substitution	Pathogenic
ATM	NM_000051.3:c.875C>T	p.Pro292Leu	substitution	Pathogenic
ATM	NM_000051.3:c.5228C>T	p.Thr1743Ile	substitution	Likely Pathogenic
ATM	NM_000051.3:c.8879G>A	p.Trp2960*	substitution	Pathogenic
ATM	NM_000051.3:c.6258T>G	p.Tyr2086*	substitution	Pathogenic

ATM	NM_000051.3:c.7271T>G	p.Val2424Gly	substitution	Pathogenic
ATM	NM_000051.3:c.8419-2A>G	Splice site	substitution	Likely Pathogenic
ATM	NM_000051.3:c.8786+1G>A	Splice site	substitution	Pathogenic
ATM	NM_000051.3:c.5006-1G>A	Splice site	substitution	Likely Pathogenic
ATM	NM_000051.3:c.2921+1G>A	Splice site	substitution	Pathogenic
ATM	NM_000051.3:c.186-2A>G	Splice site	substitution	Likely Pathogenic
ATM	NM_000051.3:c.5497-2A>C	Splice site	substitution	Pathogenic
ATP7B	NM_000053.3:c.-157-?_51+?del	Deletion (Exon 1)	cnv	Pathogenic
ATP7B	NM_000053.3:c.3402del	p.Ala1135Glnfs	indel	Pathogenic
ATP7B	NM_000053.3:c.19_20del	p.Gln7Aspfs	indel	Pathogenic
ATP7B	NM_000053.3:c.1745_1746del	p.Ile582Argfs	indel	Pathogenic
ATP7B	NM_000053.3:c.2304dup	p.Met769Hisfs	indel	Pathogenic
ATP7B	NM_000053.3:c.2781del	p.Phe927Leufs	indel	Pathogenic
ATP7B	NM_000053.3:c.956del	p.Pro319Hisfs	indel	Pathogenic
ATP7B	NM_000053.3:c.2009_2015del	p.Tyr670*	indel	Pathogenic
ATP7B	NM_000053.3:c.3649_3654del	p.Val1217_Leu1218del	indel	Likely Pathogenic
ATP7B	NM_000053.3:c.2532del	p.Val845Serfs	indel	Pathogenic
ATP7B	NM_000053.3:c.51+4A>T	Intronic	substitution	Pathogenic
ATP7B	NM_000053.3:c.2122-3C>T	Intronic	substitution	Likely Pathogenic
ATP7B	NM_000053.3:c.-676A>G	Non-coding	substitution	Likely Pathogenic
ATP7B	NM_000053.3:c.3007G>A	p.Ala1003Thr	substitution	Pathogenic
ATP7B	NM_000053.3:c.3451C>T	p.Arg1151Cys	substitution	Likely Pathogenic
ATP7B	NM_000053.3:c.3955C>T	p.Arg1319*	substitution	Pathogenic
ATP7B	NM_000053.3:c.1847G>A	p.Arg616Gln	substitution	Pathogenic
ATP7B	NM_000053.3:c.2333G>T	p.Arg778Leu	substitution	Pathogenic
ATP7B	NM_000053.3:c.122A>G	p.Asn41Ser	substitution	Pathogenic
ATP7B	NM_000053.3:c.3191A>C	p.Glu1064Ala	substitution	Pathogenic
ATP7B	NM_000053.3:c.3517G>A	p.Glu1173Lys	substitution	Pathogenic
ATP7B	NM_000053.3:c.3796G>A	p.Gly1266Arg	substitution	Pathogenic
ATP7B	NM_000053.3:c.2071G>A	p.Gly691Arg	substitution	Pathogenic
ATP7B	NM_000053.3:c.2128G>A	p.Gly710Ser	substitution	Pathogenic
ATP7B	NM_000053.3:c.2605G>A	p.Gly869Arg	substitution	Likely Pathogenic
ATP7B	NM_000053.3:c.3207C>A	p.His1069Gln	substitution	Pathogenic
ATP7B	NM_000053.3:c.2383C>T	p.Leu795Phe	substitution	Pathogenic
ATP7B	NM_000053.3:c.1934T>G	p.Met645Arg	substitution	Pathogenic
ATP7B	NM_000053.3:c.2305A>G	p.Met769Val	substitution	Pathogenic
ATP7B	NM_000053.3:c.2975C>T	p.Pro992Leu	substitution	Pathogenic
ATP7B	NM_000053.3:c.4088C>T	p.Ser1363Phe	substitution	Pathogenic
ATP7B	NM_000053.3:c.2924C>A	p.Ser975Tyr	substitution	Pathogenic
ATP7B	NM_000053.3:c.4058G>A	p.Trp1353*	substitution	Pathogenic
ATP7B	NM_000053.3:c.3316G>A	p.Val1106Ile	substitution	Pathogenic
ATP7B	NM_000053.3:c.2668G>A	p.Val890Met	substitution	Likely Pathogenic
AXIN2	NM_004655.3:c.1908-2A>G	Splice site	substitution	Likely Pathogenic
BAP1	NM_004656.3:c.1383dup	p.Pro462Serfs	indel	Pathogenic
BAP1	NM_004656.3:c.783+2T>C	Splice site	substitution	Likely Pathogenic
BARD1	NM_000465.3:c.1315-?_1568+?del	Deletion (Exons 5-6)	cnv	Pathogenic
BARD1	NM_000465.3:c.1904-3360_2020del	Partial Deletion (Exons 10-11)	cnv	Likely Pathogenic
BARD1	NM_000465.3:c.1865_1903+274del	Partial Deletion (Exon 9)	cnv	Likely Pathogenic
BARD1	NM_000465.3:c.69_70delins25	p.Ala25Glyfs	indel	Pathogenic
BARD1	NM_000465.3:c.2229dup	p.Asn744*	indel	Likely Pathogenic
BARD1	NM_000465.3:c.176_177del	p.Glu59Alafs	indel	Pathogenic
BARD1	NM_000465.3:c.1935_1954dup	p.Glu652Valfs	indel	Pathogenic
BARD1	NM_000465.3:c.1996C>T	p.Gln666*	substitution	Likely Pathogenic
BARD1	NM_000465.3:c.1212C>G	p.Tyr404*	substitution	Pathogenic
BRCA1	NM_007294.3:c.5333-?_5406+?del	Deletion (Exon 21)	cnv	Pathogenic
BRCA1	NM_007294.3:c.-232-?_5467+?del	Deletion (Exons 1-22)	cnv	Pathogenic
BRCA1	NM_007294.3:c.4186-?_4675+?del	Deletion (Exons 12-14)	cnv	Pathogenic
BRCA1	NM_007294.3:c.4358-?_5277+?del	Deletion (Exons 13-19)	cnv	Pathogenic
BRCA1	NM_007294.3:c.548-?_4185+?del	Deletion (Exons 8-11)	cnv	Pathogenic
BRCA1	NM_007294.3:c.4186-?_4357+?dup	Gain (Exon 12)	cnv	Pathogenic
BRCA1	NM_007294.3:c.2071del	p.Arg691Aspfs	indel	Pathogenic

BRCA1	NM_007294.3:c.2989_2990dup	p.Asn997Lysfs	indel	Pathogenic
BRCA1	NM_007294.3:c.3485del	p.Asp1162Valfs	indel	Pathogenic
BRCA1	NM_007294.3:c.676del	p.Cys226Valfs	indel	Pathogenic
BRCA1	NM_007294.3:c.3331_3334del	p.Gln1111Asnfs	indel	Pathogenic
BRCA1	NM_007294.3:c.5266dupC	p.Gln1756Profs	indel	Pathogenic
BRCA1	NM_007294.3:c.4035del	p.Glu1346Lysfs	indel	Pathogenic
BRCA1	NM_007294.3:c.66dup	p.Glu23Argfs	indel	Pathogenic
BRCA1	NM_007294.3:c.68_69delAG	p.Glu23Valfs	indel	Pathogenic
BRCA1	NM_007294.3:c.3228_3229del	p.Gly1077Alafs	indel	Pathogenic
BRCA1	NM_007294.3:c.2517_2518del	p.His839Glnfs	indel	Pathogenic
BRCA1	NM_007294.3:c.5470_5477del	p.Ile1824Aspfs	indel	Pathogenic
BRCA1	NM_007294.3:c.432_433del	p.Pro145Phefs	indel	Pathogenic
BRCA1	NM_007294.3:c.2926_2941dup	p.Pro981Glnfs	indel	Pathogenic
BRCA1	NM_007294.3:c.2865del	p.Ser956Leufs	indel	Pathogenic
BRCA1	NM_007294.3:c.1961dup	p.Tyr655Valfs	indel	Pathogenic
BRCA1	NM_007294.3:c.697_698del	p.Val233Asnfs	indel	Pathogenic
BRCA1	NM_007294.3:c.2T>C	Initiator codon	substitution	Pathogenic
BRCA1	NM_007294.3:c.5503C>T	p.Arg1835*	substitution	Pathogenic
BRCA1	NM_007294.3:c.191G>A	p.Cys64Tyr	substitution	Pathogenic
BRCA1	NM_007294.3:c.1687C>T	p.Gln563*	substitution	Pathogenic
BRCA1	NM_007294.3:c.4057G>T	p.Glu1353*	substitution	Pathogenic
BRCA1	NM_007294.3:c.427G>T	p.Glu143*	substitution	Pathogenic
BRCA1	NM_007294.3:c.5117G>A	p.Gly1706Glu	substitution	Pathogenic
BRCA1	NM_007294.3:c.4508C>A	p.Ser1503*	substitution	Pathogenic
BRCA1	NM_007294.3:c.5346G>A	p.Trp1782*	substitution	Pathogenic
BRCA1	NM_007294.3:c.5152+1G>T	Splice site	substitution	Pathogenic
BRCA1	NM_007294.3:c.4485-1G>A	Splice site	substitution	Likely Pathogenic
BRCA1	NM_007294.3:c.5277+1G>A	Splice site	substitution	Pathogenic
BRCA2	NM_007294.3:c.425+415_4780dup	Gain (Exons 5-11)	cnv	Pathogenic
BRCA2	NM_000059.3:c.2808_2811del	p.Ala938Profs	indel	Pathogenic
BRCA2	NM_000059.3:c.5238dup	p.Asn1747*	indel	Pathogenic
BRCA2	NM_000059.3:c.5350_5351del	p.Asn1784Hisfs	indel	Pathogenic
BRCA2	NM_000059.3:c.6408_6414del	p.Asn2137Lysfs	indel	Pathogenic
BRCA2	NM_000059.3:c.1265del	p.Asn422Ilefs	indel	Pathogenic
BRCA2	NM_000059.3:c.2836_2837del	p.Asp946Phefs	indel	Pathogenic
BRCA2	NM_000059.3:c.9699_9702del	p.Cys3233Trpfs	indel	Pathogenic
BRCA2	NM_000059.3:c.4284dup	p.Gln1429Serfs	indel	Pathogenic
BRCA2	NM_000059.3:c.6024dup	p.Gln2009Alafs	indel	Pathogenic
BRCA2	NM_000059.3:c.6468_6469del	p.Gln2157Ilefs	indel	Pathogenic
BRCA2	NM_000059.3:c.9891_9894dup	p.Gln3299Ilefs	indel	Pathogenic
BRCA2	NM_000059.3:c.5722_5723del	p.Leu1908Argfs	indel	Pathogenic
BRCA2	NM_000059.3:c.6275_6276del	p.Leu2092Profs	indel	Pathogenic
BRCA2	NM_000059.3:c.6757_6758del	p.Leu2253Phefs	indel	Pathogenic
BRCA2	NM_000059.3:c.3170_3174delAGAAA	p.Lys1057Thrfs	indel	Pathogenic
BRCA2	NM_000059.3:c.469_470delAA	p.Lys157Valfs	indel	Pathogenic
BRCA2	NM_000059.3:c.6486_6489del	p.Lys2162Asnfs	indel	Pathogenic
BRCA2	NM_000059.3:c.3545_3546del	p.Phe1182*	indel	Pathogenic
BRCA2	NM_000059.3:c.2672dup	p.Phe892Leufs	indel	Pathogenic
BRCA2	NM_000059.3:c.6998dup	p.Pro2334Thrfs	indel	Pathogenic
BRCA2	NM_000059.3:c.3744_3747del	p.Ser1248Argfs	indel	Pathogenic
BRCA2	NM_000059.3:c.5828del	p.Ser1943Leufs	indel	Pathogenic
BRCA2	NM_000059.3:c.5946delT	p.Ser1982Argfs	indel	Pathogenic
BRCA2	NM_000059.3:c.3199del	p.Thr1067Leufs	indel	Pathogenic
BRCA2	NM_000059.3:c.4133_4136del	p.Thr1378Argfs	indel	Pathogenic
BRCA2	NM_000059.3:c.9097dup	p.Thr3033Asnfs	indel	Pathogenic
BRCA2	NM_000059.3:c.9253dup	p.Thr3085Asnfs	indel	Pathogenic
BRCA2	NM_000059.3:c.2716dup	p.Thr906Asnfs	indel	Pathogenic
BRCA2	NM_000059.3:c.5130_5133del	p.Tyr1710*	indel	Pathogenic
BRCA2	NM_000059.3:c.6641dup	p.Tyr2215Leufs	indel	Pathogenic
BRCA2	NM_000059.3:c.3847_3848del	p.Val1283Lysfs	indel	Pathogenic
BRCA2	NM_000059.3:c.156_157insAlu	Splicing	indel	Pathogenic

BRCA2	NM_000059.3:c.3G>A	Initiator codon	substitution	Pathogenic
BRCA2	NM_000059.3:c.7007G>A	p.Arg2336His	substitution	Pathogenic
BRCA2	NM_000059.3:c.7007G>C	p.Arg2336Pro	substitution	Pathogenic
BRCA2	NM_000059.3:c.7976G>A	p.Arg2659Lys	substitution	Pathogenic
BRCA2	NM_000059.3:c.5864C>A	p.Ser1955*	substitution	Pathogenic
BRCA2	NM_000059.3:c.1103C>A	p.Ser368*	substitution	Pathogenic
BRCA2	NM_000059.3:c.8909G>A	p.Trp2970*	substitution	Pathogenic
BRCA2	NM_000059.3:c.5286T>G	p.Tyr1762*	substitution	Pathogenic
BRCA2	NM_000059.3:c.8488-1G>A	Splice site	substitution	Likely Pathogenic
BRCA2	NM_000059.3:c.-39-1G>C	Splice site	substitution	Likely Pathogenic
BRIP1	NM_032043.2:c.2010dup	p.Glu671*	indel	Pathogenic
BRIP1	NM_032043.2:c.1442_1443dup	p.Ile482Valfs	indel	Pathogenic
BRIP1	NM_032043.2:c.1510dup	p.Ile504Asnfs	indel	Pathogenic
BRIP1	NM_032043.2:c.2992_2995del	p.Lys998Glufs	indel	Pathogenic
BRIP1	NM_032043.2:c.3401del	p.Pro1134Leufs	indel	Likely Pathogenic
BRIP1	NM_032043.2:c.1853_1854insG	p.Pro619Thrfs	indel	Pathogenic
BRIP1	NM_032043.2:c.2684_2687del	p.Ser895*	indel	Pathogenic
BRIP1	NM_032043.2:c.1045G>C	p.Ala349Pro	substitution	Likely Pathogenic
BRIP1	NM_032043.2:c.1315C>T	p.Arg439*	substitution	Pathogenic
BRIP1	NM_032043.2:c.2392C>T	p.Arg798*	substitution	Pathogenic
BRIP1	NM_032043.2:c.1372G>T	p.Glu458*	substitution	Pathogenic
BRIP1	NM_032043.2:c.161T>G	p.Leu54*	substitution	Pathogenic
BRIP1	NM_032043.2:c.3167C>G	p.Ser1056*	substitution	Likely Pathogenic
BRIP1	NM_032043.2:c.1694C>A	p.Ser565*	substitution	Pathogenic
BRIP1	NM_032043.2:c.1343G>A	p.Trp448*	substitution	Pathogenic
BRIP1	NM_032043.2:c.2400C>G	p.Tyr800*	substitution	Pathogenic
BRIP1	NM_032043.2:c.1340+1G>A	Splice site	substitution	Likely Pathogenic
CACNA1S	NM_000069.2:c.5227-2del	Splice site	indel	Likely Pathogenic
CACNA1S	NM_000069.2:c.19C>T	p.Gln7*	substitution	Pathogenic
CACNA1S	NM_000069.2:c.1948+1G>A	Splice site	substitution	Likely Pathogenic
CACNA1S	NM_000069.2:c.4442-2A>G	Splice site	substitution	Likely Pathogenic
CACNA1S	NM_000069.2:c.2854-2A>C	Splice site	substitution	Likely Pathogenic
CACNA1S	NM_000069.2:c.3525+1G>A	Splice site	substitution	Likely Pathogenic
CASQ2	NM_001232.3:c.475G>T	p.Glu159*	substitution	Pathogenic
CASQ2	NM_001232.3:c.856G>T	p.Glu286*	substitution	Pathogenic
CAV3	NM_033337.2:c.277G>A	p.Ala93Thr	substitution	Pathogenic
CAV3	NM_033337.2:c.294C>A	p.Cys98*	substitution	Likely Pathogenic
CDH1	NM_004360.3:c.1973dup	p.Leu658Phefs	indel	Pathogenic
CDH1	NM_004360.3:c.2287G>T	p.Glu763*	substitution	Pathogenic
CDKN2A (p16INK4a)	NM_000077.4:c.225_243del	p.Ala76Cysfs	indel	Pathogenic
CDKN2A (p16INK4a)	NM_000077.4:c.335_337dup	p.Arg112dup	indel	Likely Pathogenic
CDKN2A (p16INK4a)	NM_000077.4:c.189del	p.Leu64Cysfs	indel	Pathogenic
CDKN2A (p16INK4a)	NM_000077.4:c.176T>G	p.Val59Gly	substitution	Pathogenic
CHEK2	NM_007194.3:c.-6-?_319+?del	Deletion (Exon 2)	cnv	Pathogenic
CHEK2	NM_007194.3:c.909-?_1095+?del	Deletion (Exons 9-10)	cnv	Pathogenic
CHEK2	NM_007194.3:c.847-?_908+?dup	Gain (Exon 8)	cnv	Likely Pathogenic
CHEK2	NM_007194.3:c.320-?_592+?dup	Gain (Exons 3-4)	cnv	Likely Pathogenic
CHEK2	NM_007194.3:c.846+4_846+7del	Intronic	indel	Likely Pathogenic
CHEK2	NM_007194.3:c.655del	p.Glu219Asnfs	indel	Pathogenic
CHEK2	NM_007194.3:c.1368dupA	p.Glu457Argfs	indel	Pathogenic
CHEK2	NM_007194.3:c.1371_1372del	p.Lys458Serfs	indel	Pathogenic
CHEK2	NM_007194.3:c.629_632del	p.Ser210Phefs	indel	Pathogenic
CHEK2	NM_007194.3:c.1263delT	p.Ser422Valfs	indel	Pathogenic
CHEK2	NM_007194.3:c.152_155dup	p.Ser53Valfs	indel	Pathogenic
CHEK2	NM_007194.3:c.1100delC	p.Thr367Metfs	indel	Pathogenic
CHEK2	NM_007194.3:c.277del	p.Trp93Glyfs	indel	Pathogenic
CHEK2	NM_007194.3:c.1188del	p.Val397Phefs	indel	Pathogenic
CHEK2	NM_007194.3:c.349A>G	p.Arg117Gly	substitution	Likely Pathogenic
CHEK2	NM_007194.3:c.433C>T	p.Arg145Trp	substitution	Likely Pathogenic
CHEK2	NM_007194.3:c.1555C>T	p.Arg519*	substitution	Likely Pathogenic
CHEK2	NM_007194.3:c.190G>A	p.Glu64Lys	substitution	Likely Pathogenic

CHEK2	NM_007194.3:c.470T>C	p.Ile157Thr	substitution	Pathogenic (low penetrance)
CHEK2	NM_007194.3:c.707T>C	p.Leu236Pro	substitution	Likely Pathogenic
CHEK2	NM_007194.3:c.1283C>T	p.Ser428Phe	substitution	Pathogenic (low penetrance)
CHEK2	NM_007194.3:c.1232G>A	p.Trp411*	substitution	Pathogenic
CHEK2	NM_007194.3:c.444+1G>A	Splice site	substitution	Pathogenic
CHEK2	NM_007194.3:c.908+2T>C	Splice site	substitution	Likely Pathogenic
CHEK2	NM_007194.3:c.1462-1G>A	Splice site	substitution	Likely Pathogenic
COL3A1	NM_000090.3:c.2248G>C	p.Gly750Arg	substitution	Likely Pathogenic
COL3A1	NM_000090.3:c.852+2T>C	Splice site	substitution	Likely Pathogenic
CRYAB	NM_001885.2:c.343del	p.Ser115Profs	indel	Pathogenic
DES	NM_001927.3:c.-86-?_*749+?del	Deletion (Entire coding sequence)	cnv	Pathogenic
DES	NM_001927.3:c.1A>G	Initiator codon	substitution	Likely Pathogenic
DES	NM_001927.3:c.322G>T	p.Glu108*	substitution	Pathogenic
DES	NM_001927.3:c.1371+1G>A	Splice site	substitution	Likely Pathogenic
DMD	NM_004006.2:c.6913-?_7098+?del	Deletion (Exon 48)	cnv	Pathogenic
DMD	NM_004006.2:c.94-?_960+?del	Deletion (Exons 3-9)	cnv	Likely Pathogenic
DMD	NM_004006.2:c.7310-?_7660+?del	Deletion (Exons 51-52)	cnv	Pathogenic
DMD	NM_004006.2:c.8028-?_8217+?dup	Gain (Exon 55)	cnv	Likely Pathogenic
DMD	NM_004006.2:c.6474del	p.Val2159Serfs	indel	Pathogenic
DSG2	NM_001943.3:c.2620del	p.Thr874Leufs	indel	Pathogenic
DSG2	NM_001943.3:c.1672C>T	p.Gln558*	substitution	Pathogenic
DSP	NM_004415.2:c.5671_*1792delins16	p.Glu1891Argfs	cnv	Pathogenic
DSP	NM_004415.2:c.2870_2874del	p.Ser957*	indel	Pathogenic
DSP	NM_004415.2:c.5212C>T	p.Arg1738*	substitution	Pathogenic
DSP	NM_004415.2:c.250C>T	p.Arg84*	substitution	Pathogenic
DSP	NM_004415.2:c.85G>T	p.Glu29*	substitution	Pathogenic
DSP	NM_004415.2:c.2437-1G>C	Splice site	substitution	Likely Pathogenic
ENG	NM_000118.3:c.511C>T	p.Arg171*	substitution	Pathogenic
EPCAM	NM_002354.2:c.-358-?_76+?del	Deletion (Exon 1)	cnv	Pathogenic
F2	NM_000506.3:c.*97G>A	Non-coding	substitution	Pathogenic (low penetrance)
F5	NM_000130.4:c.1601G>A	p.Arg534Gln	substitution	Pathogenic
FH	NM_000143.3:c.1431_1433dupAAA	p.Lys477dup	indel	Likely Pathogenic
FH	NM_000143.3:c.782G>T	p.Arg261Ile	substitution	Likely Pathogenic
FH	NM_000143.3:c.194A>G	p.Asp65Gly	substitution	Likely Pathogenic
FH	NM_000143.3:c.1189G>A	p.Gly397Arg	substitution	Pathogenic
FH	NM_000143.3:c.521C>G	p.Pro174Arg	substitution	Pathogenic
FH	NM_000143.3:c.1093A>G	p.Ser365Gly	substitution	Pathogenic
FLCN	NM_144997.5:c.-504-?_-228+?del	Deletion (Exon 1)	cnv	Pathogenic
FLCN	NM_144997.5:c.584del	p.Gly195Glufs	indel	Pathogenic
FLCN	NM_144997.5:c.521_527del	p.Thr174Argfs	indel	Pathogenic
FLNC	NM_001458.4:c.4581-?_4927+?del	Deletion (Exons 27-28)	cnv	Pathogenic
FLNC	NM_001458.4:c.2084del	p.Arg695Leufs	indel	Pathogenic
FLNC	NM_001458.4:c.4882_4886delinsGCT	p.Ile1628Alafs	indel	Pathogenic
FLNC	NM_001458.4:c.4926_4927insACGTCACA	p.Val1643Thrfs	indel	Pathogenic
FLNC	NM_001458.4:c.2119C>T	p.Gln707*	substitution	Pathogenic
FLNC	NM_001458.4:c.3791-1G>C	Splice site	substitution	Pathogenic
GLA	NM_000169.2:c.640-801G>A	Intronic	substitution	Pathogenic
GLA	NM_000169.2:c.335G>A	p.Arg112His	substitution	Pathogenic
GLA	NM_000169.2:c.644A>G	p.Asn215Ser	substitution	Pathogenic
HFE	NM_000410.3:c.762del	p.Asp255Thrfs	indel	Pathogenic
HFE	NM_000410.3:c.211C>T	p.Arg71*	substitution	Pathogenic
HFE	NM_000410.3:c.845G>A	p.Cys282Tyr	substitution	Pathogenic (low penetrance)
HFE	NM_000410.3:c.502G>T	p.Glu168*	substitution	Pathogenic
HFE	NM_000410.3:c.187C>G	p.His63Asp	substitution	Pathogenic (low penetrance)
HFE	NM_000410.3:c.341-1G>A	Splice site	substitution	Likely Pathogenic
HJV	NM_213653.3:c.959G>T	p.Gly320Val	substitution	Pathogenic
HJV	NM_213653.3:c.1097T>A	p.Leu366*	substitution	Pathogenic
HOXB13	NM_006361.5:c.251G>A	p.Gly84Glu	substitution	Increased Risk Allele
JUP	NM_002230.2:c.532_542del	p.Ala178Leufs	indel	Pathogenic
JUP	NM_002230.2:c.902A>G	p.Glu301Gly	substitution	Likely Pathogenic
JUP	NM_002230.2:c.545C>A	p.Ser182*	substitution	Pathogenic

KCNE1	NM_000219.5:c.292C>T	p.Arg98Trp	substitution	Likely Pathogenic
KCNE1	NM_000219.5:c.226G>A	p.Asp76Asn	substitution	Pathogenic
KCNE1	NM_000219.5:c.172_177delinsCCCCCT	p.Thr58_Leu59delinsProPro	substitution	Likely Pathogenic
KCNH2	NM_172057.2:c.2692_2692+1insACACGG	Splice site	indel	Likely Pathogenic
KCNQ1	NM_000218.2:c.1893dup	p.Arg632Glnfs	indel	Pathogenic
KCNQ1	NM_000218.2:c.524_534del	p.Leu175Argfs	indel	Pathogenic
KCNQ1	NM_000218.2:c.905C>T	p.Ala302Val	substitution	Likely Pathogenic
KCNQ1	NM_000218.2:c.776G>A	p.Arg259His	substitution	Pathogenic
KCNQ1	NM_000218.2:c.502G>A	p.Gly168Arg	substitution	Pathogenic
KCNQ1	NM_000218.2:c.1085A>G	p.Lys362Arg	substitution	Pathogenic
KCNQ1	NM_000218.2:c.153C>G	p.Tyr51*	substitution	Pathogenic
LDLR	NM_000527.4:c.-187-?_67+?del	Deletion (Exon 1)	cnv	Pathogenic
LDLR	NM_000527.4:c.1846-?_2140+?del	Deletion (Exons 13-14)	cnv	Pathogenic
LDLR	NM_000527.4:c.2390-?_*2514+?del	Deletion (Exons 17-18)	cnv	Pathogenic
LDLR	NM_000527.4:c.1187-?_2140+?del	Deletion (Exons 9-14)	cnv	Pathogenic
LDLR	NM_000527.4:c.2416dup	p.Val806Glyfs	indel	Pathogenic
LDLR	NM_000527.4:c.-152C>T	Non-coding	substitution	Pathogenic
LDLR	NM_000527.4:c.1195G>A	p.Ala399Thr	substitution	Likely Pathogenic
LDLR	NM_000527.4:c.1246C>T	p.Arg416Trp	substitution	Pathogenic
LDLR	NM_000527.4:c.1721G>A	p.Arg574His	substitution	Likely Pathogenic
LDLR	NM_000527.4:c.1898G>A	p.Arg633His	substitution	Likely Pathogenic
LDLR	NM_000527.4:c.241C>T	p.Arg81Cys	substitution	Likely Pathogenic
LDLR	NM_000527.4:c.798T>A	p.Asp266Glu	substitution	Pathogenic
LDLR	NM_000527.4:c.912C>G	p.Asp304Glu	substitution	Pathogenic
LDLR	NM_000527.4:c.81C>G	p.Cys27Trp	substitution	Pathogenic
LDLR	NM_000527.4:c.1135T>C	p.Cys379Arg	substitution	Pathogenic
LDLR	NM_000527.4:c.2029T>C	p.Cys677Arg	substitution	Pathogenic
LDLR	NM_000527.4:c.301G>A	p.Glu101Lys	substitution	Pathogenic
LDLR	NM_000527.4:c.337G>A	p.Glu113Lys	substitution	Likely Pathogenic
LDLR	NM_000527.4:c.1003G>A	p.Gly335Ser	substitution	Likely Pathogenic
LDLR	NM_000527.4:c.1027G>A	p.Gly343Ser	substitution	Pathogenic
LDLR	NM_000527.4:c.1775G>A	p.Gly592Glu	substitution	Pathogenic
LDLR	NM_000527.4:c.1747C>T	p.His583Tyr	substitution	Pathogenic
LDLR	NM_000527.4:c.1745T>C	p.Leu582Pro	substitution	Likely Pathogenic
LDLR	NM_000527.4:c.2054C>T	p.Pro685Leu	substitution	Pathogenic
LDLR	NM_000527.4:c.2096C>T	p.Pro699Leu	substitution	Likely Pathogenic
LDLR	NM_000527.4:c.858C>A	p.Ser286Arg	substitution	Likely Pathogenic
LDLR	NM_000527.4:c.11G>A	p.Trp4*	substitution	Pathogenic
LDLR	NM_000527.4:c.2389G>A	p.Val797Met	substitution	Pathogenic
LDLRAP1	Deletion	Entire coding sequence	cnv	Pathogenic
LDLRAP1	NM_015627.2:c.65G>A	p.Trp22*	substitution	Pathogenic
LMNA	NM_170707.3:c.992G>A	p.Arg331Gln	substitution	Pathogenic
LMNA	NM_170707.3:c.1130G>A	p.Arg377His	substitution	Pathogenic
LMNA	NM_170707.3:c.1580G>A	p.Arg527His	substitution	Pathogenic
MEN1	NM_130799.2:c.914del	p.Gly305Alafs	indel	Pathogenic
MITF	NM_000248.3:c.952G>A	p.Glu318Lys	substitution	Pathogenic
MLH1	NM_000249.3:c.1852_1854del	p.Lys618del	indel	Pathogenic
MLH1	NM_000249.3:c.116+5G>A	Intronic	substitution	Likely Pathogenic
MLH1	NM_000249.3:c.2041G>A	p.Ala681Thr	substitution	Pathogenic
MLH1	NM_000249.3:c.2142G>A	p.Trp714*	substitution	Pathogenic
MLH1	NM_000249.3:c.1517T>C	p.Val506Ala	substitution	Likely Pathogenic
MLH1	NM_000249.3:c.1896+1G>A	Splice site	substitution	Pathogenic
MSH2	NM_000251.2:c.1151dup	p.Asp386Argfs	indel	Pathogenic
MSH2	NM_000251.2:c.2633_2634del	p.Glu878Alafs	indel	Pathogenic
MSH2	NM_000251.2:c.190del	p.Ile64Serfs	indel	Pathogenic
MSH2	NM_000251.2:c.1571G>C	p.Arg524Pro	substitution	Pathogenic
MSH2	NM_000251.2:c.2635-1G>T	Splice site	substitution	Likely Pathogenic
MSH3	NM_002439.4:c.1654-?_1763+?del	Deletion (Exon 12)	cnv	Pathogenic
MSH3	NM_002439.4:c.2668_2671del	p.Arg890*	indel	Pathogenic
MSH3	NM_002439.4:c.1648_1649dup	p.Asn550Lysfs	indel	Pathogenic
MSH3	NM_002439.4:c.1035del	p.Leu347*	indel	Pathogenic

MSH3	NM_002439.4:c.978_984del	p.Phe326Leufs	indel	Pathogenic
MSH3	NM_002439.4:c.1417dup	p.Thr473Asnfs	indel	Pathogenic
MSH3	NM_002439.4:c.2179C>T	p.Arg727*	substitution	Pathogenic
MSH3	NM_002439.4:c.697G>T	p.Glu233*	substitution	Pathogenic
MSH3	NM_002439.4:c.2686G>T	p.Gly896*	substitution	Pathogenic
MSH3	NM_002439.4:c.2663C>G	p.Ser888*	substitution	Pathogenic
MSH3	NM_002439.4:c.2319-1G>A	Splice site	substitution	Pathogenic
MSH3	NM_002439.4:c.2655+1G>A	Splice site	substitution	Likely Pathogenic
MSH6	NM_000179.2:c.3959_3962del	p.Ala1320Glufs	indel	Pathogenic
MSH6	NM_000179.2:c.1519dup	p.Arg507Lysfs	indel	Pathogenic
MSH6	NM_000179.2:c.3964_3980dup	p.Asn1327Lysfs	indel	Pathogenic
MSH6	NM_000179.2:c.706_707del	p.Gln236Argfs	indel	Pathogenic
MSH6	NM_000179.2:c.3744_3773del	p.His1248_Ser1257del	indel	Pathogenic
MSH6	NM_000179.2:c.3984_3987dup	p.Leu1330Valfs	indel	Pathogenic
MSH6	NM_000179.2:c.2677_2678del	p.Leu893Alafs	indel	Pathogenic
MSH6	NM_000179.2:c.3261dup	p.Phe1088Leufs	indel	Pathogenic
MSH6	NM_000179.2:c.2269_2270del	p.Thr757Profs	indel	Pathogenic
MSH6	NM_000179.2:c.3226C>T	p.Arg1076Cys	substitution	Pathogenic
MSH6	NM_000179.2:c.3227G>A	p.Arg1076His	substitution	Likely Pathogenic
MSH6	NM_000179.2:c.2731C>T	p.Arg911*	substitution	Pathogenic
MSH6	NM_000179.2:c.463A>T	p.Lys155*	substitution	Pathogenic
MSH6	NM_000179.2:c.901A>T	p.Lys301*	substitution	Pathogenic
MSH6	NM_000179.2:c.1721C>G	p.Ser574*	substitution	Pathogenic
MSH6	NM_000179.2:c.3556+2T>C	Splice site	substitution	Likely Pathogenic
MUTYH	NM_001128425.1:c.504+19_504+31del	Intronic	indel	Pathogenic
MUTYH	NM_001128425.1:c.1147delC	p.Ala385Profs	indel	Pathogenic
MUTYH	NM_001128425.1:c.1437_1439delGGA	p.Glu480del	indel	Pathogenic
MUTYH	NM_001128425.1:c.933+3A>C	Intronic	substitution	Pathogenic
MUTYH	NM_001128425.1:c.55C>T	p.Arg19*	substitution	Pathogenic
MUTYH	NM_001128425.1:c.722G>A	p.Arg241Gln	substitution	Likely Pathogenic
MUTYH	NM_001128425.1:c.734G>A	p.Arg245His	substitution	Pathogenic
MUTYH	NM_001128425.1:c.739C>T	p.Arg247*	substitution	Pathogenic
MUTYH	NM_001128425.1:c.1012C>T	p.Gln338*	substitution	Pathogenic
MUTYH	NM_001128425.1:c.1187G>A	p.Gly396Asp	substitution	Pathogenic
MUTYH	NM_001128425.1:c.884C>T	p.Pro295Leu	substitution	Likely Pathogenic
MUTYH	NM_001128425.1:c.1214C>T	p.Pro405Leu	substitution	Pathogenic
MUTYH	NM_001128425.1:c.521G>A	p.Trp174*	substitution	Pathogenic
MUTYH	NM_001128425.1:c.536A>G	p.Tyr179Cys	substitution	Pathogenic
MUTYH	NM_001128425.1:c.789-2A>C	Splice site	substitution	Likely Pathogenic
MUTYH	NM_001128425.1:c.1187-2A>G	Splice site	substitution	Pathogenic
MUTYH	NM_001128425.1:c.1477-1G>A	Splice site	substitution	Likely Pathogenic
MYBPC3	NM_000256.3:c.3628-41_3628-17del	Intronic	indel	Pathogenic
MYBPC3	NM_000256.3:c.2864_2865del	p.Pro955Argfs	indel	Pathogenic
MYBPC3	NM_000256.3:c.1504C>T	p.Arg502Trp	substitution	Pathogenic
MYBPC3	NM_000256.3:c.1828G>C	p.Asp610His	substitution	Likely Pathogenic
MYBPC3	NM_000256.3:c.1624G>C	p.Glu542Gln	substitution	Pathogenic
MYBPC3	NM_000256.3:c.1591G>C	p.Gly531Arg	substitution	Likely Pathogenic
MYBPC3	NM_000256.3:c.655G>C	p.Val219Leu	substitution	Pathogenic
MYBPC3	NM_000256.3:c.821+1G>A	Splice site	substitution	Pathogenic
MYBPC3	NM_000256.3:c.927-2A>G	Splice site	substitution	Pathogenic
MYH11	NM_001040113.1:c.-17-?_*8+?del	Deletion (Entire coding sequence)	cnv	Pathogenic
MYH11	NM_001040113.1:c.-107-?_*997+?del	Deletion (Entire coding sequence)	cnv	Pathogenic
MYH11	NM_001040113.1:c.3624_3628dup	p.Gln1210Profs	indel	Pathogenic
MYH7	NM_000257.3:c.3134G>T	p.Arg1045Leu	substitution	Pathogenic
MYH7	NM_000257.3:c.5135G>A	p.Arg1712Gln	substitution	Likely Pathogenic
MYH7	NM_000257.3:c.5134C>T	p.Arg1712Trp	substitution	Pathogenic
MYH7	NM_000257.3:c.5342G>A	p.Arg1781His	substitution	Likely Pathogenic
MYH7	NM_000257.3:c.611G>A	p.Arg204His	substitution	Pathogenic
MYH7	NM_000257.3:c.1207C>T	p.Arg403Trp	substitution	Pathogenic
MYH7	NM_000257.3:c.1988G>A	p.Arg663His	substitution	Pathogenic
MYH7	NM_000257.3:c.2080C>T	p.Arg694Cys	substitution	Likely Pathogenic

MYH7	NM_000257.3:c.2167C>T	p.Arg723Cys	substitution	Pathogenic
MYH7	NM_000257.3:c.2221G>T	p.Gly741Trp	substitution	Pathogenic
MYH7	NM_000257.3:c.1727A>G	p.His576Arg	substitution	Likely Pathogenic
MYH7	NM_000257.3:c.5561C>T	p.Thr1854Met	substitution	Likely Pathogenic
MYH7	NM_000257.3:c.5655G>A	Silent	substitution	Pathogenic
MYLK	NM_053025.3:c.3715del	p.Gln1239Argfs	indel	Pathogenic
NBN	NM_002485.4:c.897-?_2184+?del	Deletion (Exons 8-14)	cnv	Pathogenic
NBN	NM_002485.4:c.35_37+10del	Partial deletion (Exon 1)	cnv	Likely Pathogenic
NBN	NM_002485.4:c.163_171+3del	Partial deletion (Exon 2)	cnv	Likely Pathogenic
NBN	NM_002485.4:c.657_661delACAAA	p.Lys219Asnfs	indel	Pathogenic
NBN	NM_002485.4:c.698_701del	p.Lys233Serfs	indel	Pathogenic
NBN	NM_002485.4:c.156_157del	p.Ser53Cysfs	indel	Pathogenic
NBN	NM_002485.4:c.1737del	p.Val580Phefs	indel	Pathogenic
NBN	NM_002485.4:c.2140C>T	p.Arg714*	substitution	Pathogenic
NBN	NM_002485.4:c.1903A>T	p.Lys635*	substitution	Pathogenic
NBN	NM_002485.4:c.1496C>A	p.Ser499*	substitution	Pathogenic
NBN	NM_002485.4:c.481-2A>T	Splice site	substitution	Likely Pathogenic
NBN	NM_002485.4:c.2234+2T>G	Splice site	substitution	Likely Pathogenic
NF1	NM_000267.3:c.244_247del	p.Gln83*	indel	Pathogenic
NF1	NM_000267.3:c.4537C>T	p.Arg1513*	substitution	Pathogenic
NF1	NM_000267.3:c.7486C>T	p.Arg2496*	substitution	Pathogenic
NF1	NM_000267.3:c.2044C>T	p.Gln682*	substitution	Pathogenic
NF1	NM_000267.3:c.5431G>T	p.Glu1811*	substitution	Pathogenic
NF2	NM_000268.3:c.-443-?_*3798+?del	Deletion (Entire coding sequence)	cnv	Pathogenic
NF2	NM_000268.3:c.1575-?_1737+?dup	Gain (Exon 15)	cnv	Likely Pathogenic
NTHL1	NM_002528.6:c.235dup	p.Ala79Glyfs	indel	Pathogenic
NTHL1	NM_002528.6:c.484del	p.Asp162Thrfs	indel	Pathogenic
NTHL1	NM_002528.6:c.859C>T	p.Gln287*	substitution	Likely Pathogenic
NTHL1	NM_002528.6:c.268C>T	p.Gln90*	substitution	Pathogenic
NTHL1	NM_002528.6:c.806G>A	p.Trp269*	substitution	Likely Pathogenic
NTHL1	NM_002528.6:c.390C>A	p.Tyr130*	substitution	Pathogenic
NTHL1	NM_002528.6:c.139+1G>A	Splice site	substitution	Likely Pathogenic
NTHL1	NM_002528.6:c.550-1G>A	Splice site	substitution	Pathogenic
PALB2	NM_024675.3:c.3114-?_3201+?del	Deletion (Exon 11)	cnv	Pathogenic
PALB2	NM_024675.3:c.509_510del	p.Arg170Ilefs	indel	Pathogenic
PALB2	NM_024675.3:c.3179_3180ins(?)	p.Cys1060fs	indel	Pathogenic
PALB2	NM_024675.3:c.1675_1676delinsTG	p.Gln559*	indel	Pathogenic
PALB2	NM_024675.3:c.172_175del	p.Gln60Argfs	indel	Pathogenic
PALB2	NM_024675.3:c.532del	p.Glu178Asnfs	indel	Pathogenic
PALB2	NM_024675.3:c.1824dup	p.Ile609Tyrfs	indel	Pathogenic
PALB2	NM_024675.3:c.1592del	p.Leu531Cysfs	indel	Pathogenic
PALB2	NM_024675.3:c.3482_3483del	p.Phe1161Cysfs	indel	Pathogenic
PALB2	NM_024675.3:c.3456dup	p.Pro1153Thrfs	indel	Pathogenic
PALB2	NM_024675.3:c.1965del	p.Pro656Glnfs	indel	Pathogenic
PALB2	NM_024675.3:c.758dup	p.Ser254Ilefs	indel	Pathogenic
PALB2	NM_024675.3:c.899del	p.Thr300Lysfs	indel	Pathogenic
PALB2	NM_024675.3:c.1677del	p.Val560*	indel	Pathogenic
PALB2	NM_024675.3:c.2964del	p.Val989*	indel	Pathogenic
PALB2	NM_024675.3:c.2257C>T	p.Arg753*	substitution	Pathogenic
PALB2	NM_024675.3:c.196C>T	p.Gln66*	substitution	Pathogenic
PALB2	NM_024675.3:c.1010T>A	p.Leu337*	substitution	Pathogenic
PALB2	NM_024675.3:c.3113G>A	p.Trp1038*	substitution	Pathogenic
PALB2	NM_024675.3:c.3549C>A	p.Tyr1183*	substitution	Pathogenic
PALB2	NM_024675.3:c.3549C>G	p.Tyr1183*	substitution	Pathogenic
PCSK9	NM_174936.3:c.1394C>T	p.Ser465Leu	substitution	Likely Pathogenic
PKP2	NM_004572.3:c.1171-?_1378+?del	Deletion (Exon 5)	cnv	Pathogenic
PKP2	NM_004572.3:c.2293_2299+1008del	Partial deletion (Exon 11)	cnv	Likely Pathogenic
PKP2	NM_004572.3:c.1237C>T	p.Arg413*	substitution	Pathogenic
PKP2	NM_004572.3:c.1978C>T	p.Gln660*	substitution	Pathogenic
PKP2	NM_004572.3:c.1613G>A	p.Trp538*	substitution	Pathogenic
PKP2	NM_004572.3:c.337-2A>T	Splice site	substitution	Pathogenic

PKP2	NM_004572.3:c.2146-1G>C	Splice site	substitution	Pathogenic
PKP2	NM_004572.3:c.2489+1G>A	Splice site	substitution	Pathogenic
PLN	NM_002667.3:c.26_29dup	p.Ala11Leufs	indel	Pathogenic
PLN	NM_002667.3:c.26G>A	p.Arg9His	substitution	Likely Pathogenic
PLN	NM_002667.3:c.116T>G	p.Leu39*	substitution	Pathogenic
PMS2	NM_000535.5:c.24-?_163+?del	Deletion (Exon 2)	cnv	Pathogenic
PMS2	NM_000535.5:c.904-?_1144+?del	Deletion (Exons 9-10)	cnv	Pathogenic
PMS2	Deletion	Entire coding sequence	cnv	Pathogenic
PMS2	Deletion	Exons 12-15	cnv	Pathogenic
PMS2	NM_000535.5:c.862_863del	p.Gln288Valfs	indel	Pathogenic
PMS2	NM_000535.5:c.1831dup	p.Ile611Asnfs	indel	Pathogenic
PMS2	NM_000535.5:c.736_741delinsTGTGTGTGAAG	p.Pro246Cysfs	indel	Pathogenic
PMS2	NM_000535.5:c.1638_1639del	p.Ser547Argfs	indel	Pathogenic
PMS2	NM_000535.5:c.1009dup	p.Thr337Asnfs	indel	Pathogenic
PMS2	NM_000535.5:c.943C>T	p.Arg315*	substitution	Pathogenic
PMS2	NM_000535.5:c.949C>T	p.Gln317*	substitution	Pathogenic
PMS2	NM_000535.5:c.2249G>A	p.Gly750Asp	substitution	Likely Pathogenic
PMS2	NM_000535.5:c.1939A>T	p.Lys647*	substitution	Pathogenic
PMS2	NM_000535.5:c.137G>A	p.Ser46Asn	substitution	Pathogenic
PMS2	NM_000535.5:c.137G>T	p.Ser46Ile	substitution	Pathogenic
PMS2	NM_000535.5:c.2444C>T	p.Ser815Leu	substitution	Likely Pathogenic
PMS2	NM_000535.5:c.765C>A	p.Tyr255*	substitution	Pathogenic
PROC	NM_000312.3:c.1212dup	p.Pro405Alafs	indel	Pathogenic
PROC	NM_000312.3:c.925G>A	p.Ala309Thr	substitution	Pathogenic
PROC	NM_000312.3:c.631C>T	p.Arg211Trp	substitution	Pathogenic
PROC	NM_000312.3:c.659G>A	p.Arg220Gln	substitution	Likely Pathogenic
PROC	NM_000312.3:c.811C>T	p.Arg271Trp	substitution	Likely Pathogenic
PROC	NM_000312.3:c.169C>T	p.Arg57Trp	substitution	Pathogenic
PROC	NM_000312.3:c.889G>C	p.Asp297His	substitution	Pathogenic
PROC	NM_000312.3:c.41G>A	p.Trp14*	substitution	Pathogenic
PROS1	NM_000313.3:c.1155+5G>A	Intronic	substitution	Pathogenic
PROS1	NM_000313.3:c.1064G>A	p.Arg355His	substitution	Likely Pathogenic
PROS1	NM_000313.3:c.200A>C	p.Glu67Ala	substitution	Likely Pathogenic
PROS1	NM_000313.3:c.586A>G	p.Lys196Glu	substitution	Pathogenic
PROS1	NM_000313.3:c.601+1G>A	Splice site	substitution	Likely Pathogenic
RAD51C	NM_058216.2:c.572-?_*120+?del	Deletion (Exons 4-9)	cnv	Pathogenic
RAD51C	NM_058216.2:c.838-?_*120+?del	Deletion (Exons 6-9)	cnv	Pathogenic
RAD51C	NM_058216.2:c.732del	p.Ile244Metfs	indel	Pathogenic
RAD51C	NM_058216.2:c.181_182del	p.Leu61Alafs	indel	Pathogenic
RAD51C	NM_058216.2:c.50del	p.Phe17Serfs	indel	Pathogenic
RAD51C	NM_058216.2:c.93del	p.Phe32Serfs	indel	Pathogenic
RAD51C	NM_058216.2:c.904+5G>T	Intronic	substitution	Likely Pathogenic
RAD51C	NM_058216.2:c.397C>T	p.Gln133*	substitution	Pathogenic
RAD51C	NM_058216.2:c.905-2A>C	Splice site	substitution	Pathogenic
RAD51D	NM_002878.3:c.-256-?_738+?del	Deletion (Exons 1-8)	cnv	Pathogenic
RAD51D	NM_002878.3:c.896_*505del	Partial deletion (Exons 9-10)	cnv	Likely Pathogenic
RAD51D	NM_002878.3:c.363del	p.Ala122Glnfs	indel	Pathogenic
RAD51D	NM_002878.3:c.270_271dup	p.Lys91Ilefs	indel	Pathogenic
RAD51D	NM_002878.3:c.620C>T	p.Ser207Leu	substitution	Pathogenic
RAD51D	NM_002878.3:c.803G>A	p.Trp268*	substitution	Pathogenic
RAD51D	NM_002878.3:c.83-1G>A	Splice site	substitution	Likely Pathogenic
RAD51D	NM_002878.3:c.577-2A>G	Splice site	substitution	Likely Pathogenic
RB1	NM_000321.2:c.309_312del	p.Phe104Leufs	indel	Pathogenic
RB1	NM_000321.2:c.1981C>T	p.Arg661Trp	substitution	Pathogenic
RET	NM_020975.4:c.1826G>A	p.Cys609Tyr	substitution	Pathogenic
RET	NM_020975.4:c.2304G>C	p.Glu768Asp	substitution	Likely Pathogenic
RET	NM_020975.4:c.1998G>T	p.Lys666Asn	substitution	Pathogenic
RET	NM_020975.4:c.2410G>A	p.Val804Met	substitution	Pathogenic
RYR1	NM_000540.2:c.5077_5078delinsG	p.Leu1693Glyfs	indel	Pathogenic
RYR1	NM_000540.2:c.9554dup	p.Leu3186Alafs	indel	Pathogenic
RYR1	NM_000540.2:c.10960del	p.Leu3654Trpfs	indel	Pathogenic

RYR1	NM_000540.2:c.13335del	p.Phe4446Serfs	indel	Pathogenic
RYR1	NM_000540.2:c.8843del	p.Ser2948Cysfs	indel	Pathogenic
RYR1	NM_000540.2:c.4225C>T	p.Arg1409*	substitution	Pathogenic
RYR1	NM_000540.2:c.6721C>T	p.Arg2241*	substitution	Pathogenic
RYR1	NM_000540.2:c.11314C>T	p.Arg3772Trp	substitution	Pathogenic
RYR1	NM_000540.2:c.11708G>A	p.Arg3903Gln	substitution	Pathogenic
RYR1	NM_000540.2:c.1589G>A	p.Arg530His	substitution	Pathogenic
RYR1	NM_000540.2:c.1597C>T	p.Arg533Cys	substitution	Pathogenic
RYR1	NM_000540.2:c.10204T>G	p.Cys3402Gly	substitution	Pathogenic
RYR1	NM_000540.2:c.7300G>A	p.Gly2434Arg	substitution	Pathogenic
RYR1	NM_000540.2:c.14344G>A	p.Gly4782Arg	substitution	Pathogenic
RYR1	NM_000540.2:c.14645C>T	p.Thr4882Met	substitution	Pathogenic
RYR1	NM_000540.2:c.3381+1G>A	Splice site	substitution	Pathogenic
RYR1	NM_000540.2:c.8311-1G>C	Splice site	substitution	Likely Pathogenic
SCN5A	NM_198056.2:c.845G>A	p.Arg282His	substitution	Pathogenic
SCN5A	NM_198056.2:c.5302A>G	p.Ile1768Val	substitution	Pathogenic
SDHA	NM_004168.3:c.253_256dup	p.Asn86Ilefs	indel	Pathogenic
SDHA	NM_004168.3:c.667del	p.Asp223Ilefs	indel	Pathogenic
SDHA	NM_004168.3:c.688del	p.Glu230Serfs	indel	Pathogenic
SDHA	NM_004168.3:c.378del	p.Val127*	indel	Pathogenic
SDHA	NM_004168.3:c.91C>T	p.Arg31*	substitution	Pathogenic
SDHA	NM_004168.3:c.223C>T	p.Arg75*	substitution	Pathogenic
SDHA	NM_004168.3:c.1064+2T>A	Splice site	substitution	Likely Pathogenic
SDHA	NM_004168.3:c.621+1G>A	Splice site	substitution	Likely Pathogenic
SDHA	NM_004168.3:c.150+1G>A	Splice site	substitution	Likely Pathogenic
SDHAF2	NM_017841.2:c.199del	p.Arg67Glufs	indel	Pathogenic
SDHB	Deletion	Entire coding sequence	cnv	Pathogenic
SDHB	NM_003000.2:c.311delinsGG	p.Asn104Argfs	indel	Pathogenic
SDHB	NM_003000.2:c.640C>T	p.Gln214*	substitution	Pathogenic
SDHB	NM_003000.2:c.380T>G	p.Ile127Ser	substitution	Pathogenic
SDHB	NM_003000.2:c.600G>T	p.Trp200Cys	substitution	Pathogenic
SDHB	NM_003000.2:c.72+1G>T	Splice site	substitution	Pathogenic
SDHC	NM_003001.3:c.397C>T	p.Arg133*	substitution	Pathogenic
SDHC	NM_003001.3:c.148C>T	p.Arg50Cys	substitution	Likely Pathogenic
SDHD	NM_003002.3:c.242C>T	p.Pro81Leu	substitution	Pathogenic
SERPINA1	NM_000295.4:c.1108_1115delinsAAAAACA	p.Glu370Lysfs	indel	Pathogenic
SERPINA1	NM_000295.4:c.227_229delTCT	p.Phe76del	indel	Pathogenic
SERPINA1	NM_000295.4:c.187C>T	p.Arg63Cys	substitution	Likely Pathogenic
SERPINA1	NM_000295.4:c.839A>T	p.Asp280Val	substitution	Pathogenic
SERPINA1	NM_000295.4:c.863A>T	p.Glu288Val	substitution	Pathogenic (low penetrance)
SERPINA1	NM_000295.4:c.1096G>A	p.Glu366Lys	substitution	Pathogenic
SERPINA1	NM_000295.4:c.194T>C	p.Leu65Pro	substitution	Likely Pathogenic
SERPINA1	NM_000295.4:c.1177C>T	p.Pro393Ser	substitution	Pathogenic
SERPINC1	NM_000488.3:c.236G>A	p.Arg79His	substitution	Likely Pathogenic
SERPINC1	NM_000488.3:c.391C>T	p.Leu131Phe	substitution	Pathogenic
SERPINC1	NM_000488.3:c.218C>T	p.Pro73Leu	substitution	Pathogenic
SGCD	NM_000337.5:c.-43-?_192+?del	Deletion (Exons 2-3)	cnv	Pathogenic
SLC40A1	NM_014585.5:c.626C>T	p.Ser209Leu	substitution	Pathogenic
SMARCB1	NM_003073.3:c.629-?_795+?dup	Gain (Exon 6)	cnv	Pathogenic
TCAP	NM_003673.3:c.26_33dup	p.Glu12Argfs	indel	Pathogenic
TFR2	NM_003227.3:c.-41-?_473+?del	Deletion (Exons 1-3)	cnv	Pathogenic
TFR2	NM_003227.3:c.2014C>T	p.Gln672*	substitution	Pathogenic
TGFBR1	NM_004612.2:c.469C>T	p.Arg157*	substitution	Pathogenic
TNNI3	NM_000363.4:c.497C>T	p.Ser166Phe	substitution	Likely Pathogenic
TP53	NM_000546.5:c.810dup	p.Glu271*	indel	Pathogenic
TP53	NM_000546.5:c.524G>A	p.Arg175His	substitution	Pathogenic
TP53	NM_000546.5:c.542G>A	p.Arg181His	substitution	Pathogenic
TP53	NM_000546.5:c.530C>T	p.Pro177Leu	substitution	Likely Pathogenic
TP53	NM_000546.5:c.655C>T	p.Pro219Ser	substitution	Likely Pathogenic
TP53	NM_000546.5:c.380C>T	p.Ser127Phe	substitution	Likely Pathogenic
TP53	NM_000546.5:c.374C>T	p.Thr125Met	substitution	Pathogenic

TSC1	NM_000368.4:c.10C>T	p.Gln4*	substitution	Pathogenic
TSC1	NM_000368.4:c.1439-1G>A	Splice site	substitution	Likely Pathogenic
VHL	NM_000551.3:c.179_192del	p.Arg60Leufs	indel	Pathogenic
VHL	NM_000551.3:c.598C>T	p.Arg200Trp	substitution	Pathogenic
VHL	NM_000551.3:c.467A>G	p.Tyr156Cys	substitution	Likely Pathogenic
